# Lattice-based post-quantum image encryption with NDFA-driven spin-matrix transformation

**DOI:** 10.1038/s41598-026-63228-1

**Published:** 2026-07-27

**Authors:** Hafiz Muhammad Waseem, Noor Munir, Carsten Maple

**Affiliations:** 1 Warwick Manufacturing Group, The University of Warwick, Coventry, CV4 7AL UK; 2 Department of Engineering Sciences, University of Oxford, Parks Road, Oxford, OX1 3PJ UK

**Keywords:** Authenticated encryption, Image encryption, Key encapsulation mechanism, Lattice-based cryptography, Nondeterministic finite automaton, Post-quantum cryptography, Spin-matrix transformation, Engineering, Mathematics and computing

## Abstract

The increasing reliance on visual data transmission across distributed and cloud-based environments requires image encryption frameworks that provide both strong cryptographic guarantees and practical computational efficiency. Conventional image encryption schemes frequently rely on heuristic transformations whose security is primarily evaluated through statistical analyses, often lacking formal resistance against emerging post-quantum threats. A layered post-quantum image encryption framework is presented, integrating lattice-based key establishment, deterministic image scrambling, and authenticated encryption within a unified architecture. A shared secret is established using the sntrup761 key encapsulation mechanism, providing IND-CCA security under lattice-based assumptions. The derived session key is expanded using HKDF into independent keys for permutation scheduling and encryption, enabling separation between cryptographic security and image transformation layers. A nondeterministic finite-state scheduling mechanism drives spin-matrix-based scrambling to enhance spatial diffusion, while authenticated encryption with associated data ensures confidentiality and integrity through strict verification prior to image recovery. Security analysis shows that post-quantum security comes from the lattice-based key encapsulation mechanism rather than the image-transformation layer, and demonstrates robustness against adaptive adversarial models, while computational evaluation indicates linear scalability with image size. Statistical and robustness analyses further confirm strong diffusion properties and stable scrambling-layer behavior under noise and occlusion conditions. The proposed framework bridges standardized post-quantum cryptography with lightweight image-oriented transformations, providing a structured and practical approach for secure visual data transmission.

## Introduction

**Fig. 1:**
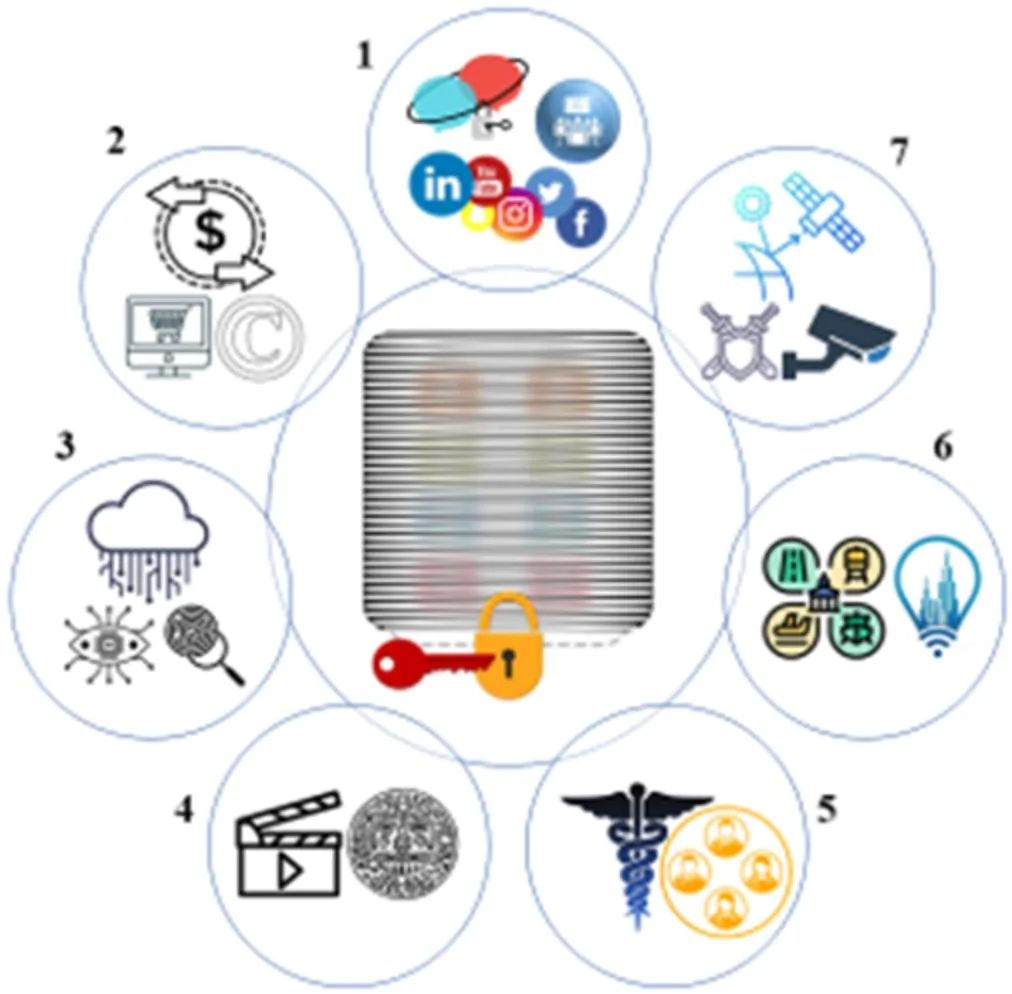
Applications of image encryption schemes: (1) Authentication systems, secure web browsing, and video conferencing; (2) E-commerce and digital watermarking; (3) Cloud computing, computer vision, and forensic evidence; (4) Art and cultural heritage and entertainment industry; (5) Medical imaging and telemedicine; (6) Smart cities and critical infrastructures; (7) Remote sensing, defense system, and CCTV surveillance.

The rapid expansion of digital technologies has led to the continuous generation, transmission, and storage of vast volumes of visual data across cloud platforms and public networks. Sensitive images generated in healthcare, finance, surveillance, defense, and industrial monitoring require strong confidentiality and integrity guarantees. Various applications of image encryption schemes are highlighted in Fig. 1. Data breaches, tampering, and unauthorized access to visual information can compromise decision-making processes and undermine trust in digital infrastructures. Consequently, image encryption has become a fundamental component of modern data security systems.

Unlike generic textual data, digital images are characterized by large data volumes, strong correlation between adjacent pixels, and substantial redundancy, which make conventional byte-oriented ciphers inefficient when applied directly and motivate dedicated image encryption designs that couple confusion and diffusion with efficient key management. This difficulty becomes greater in latency-sensitive settings such as telemedicine and real-time surveillance, where encryption must preserve confidentiality and integrity without introducing excessive computational overhead. These requirements distinguish image encryption from general-purpose data encryption and shape the design space explored in the remainder of this section.

Despite extensive research in multimedia protection, many image encryption schemes face significant challenges that limit their robustness in practical deployments. Weak key management mechanisms, insufficient resistance to adaptive adversaries, and excessive computational complexity remain persistent challenges. A secure image encryption framework must therefore satisfy several design requirements, such as (i) reliable and scalable key establishment, particularly in distributed or cloud-based environments^1,2^–^3^, (ii) resilience against classical and adaptive attack models, including brute-force, statistical, known-plaintext, and chosen-ciphertext attacks^4,5^, (iii) computational efficiency suitable for real-time or large-scale processing^6,7^, and (iv) adaptability to diverse image types and transformations such as compression, resizing, and format conversion^8^.

These challenges are further intensified by the anticipated impact of quantum computing, which threatens widely deployed classical cryptographic primitives^9,10^. The transition toward post-quantum cryptography (PQC) is therefore essential to ensure long-term security of image transmission systems.

### Related work and limitations

Numerous approaches have been proposed for image encryption, which can be broadly categorized into chaos-based methods, optimization-driven techniques, transformation-based cryptosystems, and quantum or post-quantum-inspired schemes.

1) Chaos-Based and Permutation–Diffusion Schemes.

Chaos-based encryption techniques are widely adopted due to their simplicity and ability to generate pseudorandom sequences for confusion and diffusion. These methods often rely on chaotic maps whose sensitivity to initial conditions enhances visual randomness. However, their security typically depends heavily on parameter selection and floating-point precision. As reported in^11^, improper parameter choices may introduce vulnerabilities exploitable under cryptanalytic analysis. Moreover, the iterative computation of chaotic maps can introduce computational overhead and numerical instability^12^. More recent permutation-diffusion designs have targeted throughput, for example by jointly encrypting batches of images through cross-image permutation and diffusion^13^, and by selectively encrypting semantically important regions with parallel computing to reduce overhead^14^. While these approaches improve efficiency, they continue to derive security from heuristic dynamical behavior rather than from formally analyzed cryptographic assumptions. Most importantly, many chaos-based systems lack formal cryptographic security proofs, limiting their robustness under rigorous threat models^15,16^.

2) Homomorphic and Transformation-Based Approaches.

Homomorphic encryption provides strong theoretical guarantees for performing computations over encrypted data, making it attractive for secure image processing tasks^17^. Nevertheless, such schemes impose substantial computational and storage overhead, rendering them less practical for real-time image encryption, particularly for high-resolution images and video streams^18^. Similarly, blockchain-based image protection mechanisms leverage tamper-resistant distributed ledgers^19^, but suffer from scalability limitations, high energy consumption, and dependency on the underlying blockchain infrastructure^20^.

3) Machine Learning and Metaheuristic Techniques.

Machine learning–based encryption and metaheuristic optimization methods introduce adaptive and dynamic key generation mechanisms^7,21^. While these approaches can improve diffusion properties and parameter optimization, they remain vulnerable to adversarial manipulation and parameter sensitivity^22^. Furthermore, their dependence on training datasets and convergence behavior may limit deterministic reproducibility and real-time applicability. Cellular automata (CA)-based schemes have also been explored due to their parallelism and structural complexity^23^. However, CA-based methods often exhibit limited key spaces and high sensitivity to initial states, which may weaken resistance against exhaustive or structured attacks, especially under quantum threat models.

4) Quantum and Post-Quantum Image Encryption.

Quantum image encryption schemes leverage principles of quantum mechanics to enhance security and key distribution^24,25^. Although promising in theory, practical implementation remains constrained by hardware limitations and high deployment costs^26,27^. Hybrid approaches combining classical cryptography with quantum-inspired mechanisms have also been investigated^28,29^, yet many lack integration with standardized post-quantum cryptographic primitives.

Overall, while existing image encryption techniques demonstrate favorable statistical properties, many do not explicitly ground their security in formally analyzed post-quantum constructions. A clear separation between image scrambling mechanisms and cryptographic hardness assumptions is often lacking. This gap motivates the need for a structured, layered framework that integrates standardized post-quantum key establishment with deterministic image transformation layers.

### Problem statement and contribution

The emergence of quantum computing poses a significant threat to classical public-key cryptography. Algorithms such as Shor’s algorithm^30^ undermine widely deployed number-theoretic schemes, necessitating the adoption of standardized post-quantum primitives. Although significant progress has been achieved in post-quantum key encapsulation mechanisms (KEM)^31^, their systematic integration into image encryption frameworks remains limited.

Existing image encryption schemes frequently emphasize statistical randomness without providing formal guarantees under adaptive chosen-ciphertext or quantum threat models^32,33^. Moreover, many designs conflate transformation mechanisms with cryptographic hardness, leading to ambiguous security interpretations.

To address these limitations, a layered post-quantum image encryption framework is proposed that explicitly separates cryptographic hardness from lightweight image scrambling operations. The proposed architecture integrates (i) a lattice-based KEM (sntrup761) to establish a quantum-resistant shared secret, (ii) a key derivation function (HKDF) to generate role-separated keys, (iii) an NDFA-controlled spin-matrix scrambling layer to enhance spatial diffusion, and (iv) an authenticated encryption with associated data (AEAD) scheme to ensure confidentiality and integrity.

By grounding security in an IND-CCA-secure post-quantum KEM and formally analyzed AEAD construction, while confining image-specific operations to a deterministic scrambling layer, the proposed framework provides structured and analyzable security guarantees suitable for practical deployment. The main contributions are summarized as follows:

1. A post-quantum image encryption framework integrating a lattice-based key encapsulation mechanism with structured spin-matrix scrambling is designed.

2) An NDFA-controlled scheduling mechanism is introduced to enhance diffusion prior to authenticated encryption.

3) A clear separation between cryptographic security primitives and lightweight image transformation layers is established.

4) Detailed algorithmic specifications are provided, and comprehensive statistical and security evaluations are conducted under adaptive chosen-ciphertext and quantum threat models.

The remainder of this paper is organized as follows. Section 2 presents the cryptographic preliminaries. Section 3 describes the proposed methodology, including encryption and decryption procedures. Section 4 provides security analysis and experimental evaluation. Section 5 concludes the paper and discusses future research directions.

## Preliminaries

This section introduces the fundamental cryptographic and mathematical primitives employed in the proposed image encryption scheme. It first describes the post-quantum KEM sntrup761, used to establish a shared secret between communicating parties under classical and quantum threat models. The theory of quantum spin matrices is then presented; these serve as lightweight, unitary confusion and diffusion primitives within the encryption process. Table 1 summarizes the cryptographic notation used throughout the paper to ensure clarity and consistency.

### Key encapsulation based on sntrup761

The sntrup761 scheme belongs to the NTRU Prime family of lattice-based cryptographic constructions. It operates over a quotient polynomial ring defined as:

1 $$\:{\mathcal{R}}_{q}=\frac{{\mathbb{Z}}_{q}\left[x\right]}{\left({x}^{761}-x-1\right)},$$

where * q* is a large prime modulus. The defining polynomial $\:{x}^{761}-x-1$ is chosen to avoid additional algebraic structure (e.g., cyclotomic rings), which reduces vulnerability to structural lattice attacks^34^. Elements of $\:{\mathcal{R}}_{q}$ are represented as polynomials of degree at most 760 with coefficients in $\:{\mathbb{Z}}_{q}$. Secret keys are sampled from distributions of small-norm polynomials, while public keys and ciphertexts incorporate controlled noise to ensure semantic security.

The sntrup761 KEM consists of three probabilistic polynomial-time algorithms:

1. *Key Generation*: $\:{p}_{k},\:{s}_{k}\leftarrow\:\mathrm{K}\mathrm{e}\mathrm{y}\mathrm{G}\mathrm{e}\mathrm{n}\left({1}^{\lambda\:}\right)$, where $\:{p}_{k}$ is the public key, $\:{s}_{k}$ is the private key, and $\:\lambda\:$ is the security parameter. Internally, $\:{s}_{k}$ contains small secret polynomials, while $\:{p}_{k}$ encodes a corresponding noisy public polynomial derived through ring operations modulo * q*.
2. *Encapsulation*: $\:\left({ct}_{kem},\:ss\right)\leftarrow\:Encaps\left({p}_{k}\right)$, where a random message polynomial is sampled and encrypted using the public key to produce the KEM ciphertext $\:{ct}_{kem}$. The shared secret * ss* is derived by hashing the message and ciphertext, ensuring uniform randomness.
3. *Decapsulation*: $\:ss\leftarrow\:Decaps\left({s}_{k},\:{ct}_{kem}\right)$, where the private key is used to recover the message polynomial. If decapsulation fails due to malformed ciphertexts, a pseudorandom value is returned instead, preventing error-based side-channel leakage.

**Table 1:** Notation used.

Symbol	Description	Symbol	Description	Symbol	Description
* M*	Plain image	* ss*	Shared secret	$\:\rho\:$	Correlation coefficient
* C*	Cipher image	$\:{k}_{aead}$	AEAD encryption key	* GSI*	Gradient Similarity Index
$\:{p}_{k},\:{s}_{k}$	Public and private keys	$\:{k}_{\mathrm{p}\mathrm{e}\mathrm{r}\mathrm{m}}$	Permutation key	$\:{I}_{ns}$	Noisy image
$\:\lambda\:$	Security parameter	$\:p\left(x\right)$	Probability of a pixel in the image	$\:{I}_{oc}$	Occluded image
$\:{ct}_{kem}$	sntrup761 ciphertext	$\:\theta\:$	Spin rotation angle		

**Key Derivation**: The shared secret * ss* produced by sntrup761 is not used directly for encryption. Instead, it is expanded using a cryptographic Key Derivation Function (KDF) as:.

2 $$\:\left({k}_{\mathrm{a}\mathrm{e}\mathrm{a}\mathrm{d}},\:{k}_{\mathrm{p}\mathrm{e}\mathrm{r}\mathrm{m}}\right)=\mathrm{H}\mathrm{K}\mathrm{D}\mathrm{F}\left(\mathrm{s}\mathrm{s}\parallel\mathrm{c}\mathrm{t}\mathrm{x}\right),$$

where $\:\mathrm{c}\mathrm{t}\mathrm{x}$ includes protocol identifiers, algorithm parameters, and image metadata. $\:{k}_{\mathrm{a}\mathrm{e}\mathrm{a}\mathrm{d}}$ is reserved for authenticated encryption and $\:{k}_{\mathrm{p}\mathrm{e}\mathrm{r}\mathrm{m}}$ drives the spin-matrix-based confusion and diffusion process. This separation ensures key independence, a standard requirement in modern cryptographic systems.

#### IND-CCA Security of sntrup761

sntrup761 achieves Indistinguishability under Adaptive Chosen-Ciphertext Attack (IND-CCA) security through a Fujisaki-Okamoto-style KEM construction^35^. Specifically.

- The encapsulation process embeds randomness and structured noise such that ciphertexts are computationally indistinguishable from random under the lattice hardness assumptions.
- The decapsulation algorithm performs implicit ciphertext validation, such that invalid ciphertexts result in pseudorandom shared secrets rather than explicit errors.
- The shared secret is derived by hashing both the decrypted message and the ciphertext, preventing adversaries from exploiting decryption oracles.

As a result, even an adversary with adaptive access to a decapsulation oracle cannot distinguish the encapsulated secret from random, satisfying IND-CCA security requirements.

### Quantum spin matrices

Quantum spin matrices represent the angular momentum properties of particles, particularly those with intrinsic angular momentum or spin. These matrices describe how a quantum system evolves and interacts under rotations^36^. To represent the spin component along axes, Pauli’s matrices form a basis for the space of $\:2\times\:2$ Hermitian matrices. The spin components along x-, y-, and z-axis are:

3 $$\:{\sigma\:}_{x}=\left[\begin{array}{cc}0&\:1\\\:1&\:0\end{array}\right],\:{\sigma\:}_{y}=\left[\begin{array}{cc}0&\:-i\\\:i&\:0\end{array}\right],\:\mathrm{a}\mathrm{n}\mathrm{d}\:{\sigma\:}_{z}=\left[\begin{array}{cc}1&\:0\\\:0&\:-1\end{array}\right].$$

The spin operators are defined as $\:{S}_{i}=\frac{\hslash\:}{2}{\sigma\:}_{i}$, where $\:i\in\:\left\{x,\:y,\:z\right\}$. These operators satisfy the angular momentum commutation relations, $\:\left[{S}_{i},{S}_{j}\right]=i\hslash\:{\epsilon\:}_{ijk}{S}_{k}$, where $\:{\epsilon\:}_{ijk}$ denotes the Levi-Civita symbol.

A finite rotation of a quantum state about axis * i* by angle $\:\theta\:$ is represented by the unitary operator as $$\:{R}_{i}\left(\theta\:\right)=\begin{array}{c}{e}^{i\theta\:\frac{{S}_{i}}{{\hslash\:}}}\end{array}$$. Substituting the Pauli matrices yields the explicit spin rotation matrices as:

4 $$\:{S}_{x}\left(\theta\:\right)=\left(\begin{array}{cc}\mathrm{cos}\left({\uptheta\:}/2\right)&\:i\mathrm{sin}\left({\uptheta\:}/2\right)\\\:i\mathrm{sin}\left({\uptheta\:}/2\right)&\:\mathrm{cos}\left({\uptheta\:}/2\right)\end{array}\right),\:{S}_{y}\left(\theta\:\right)=\left(\begin{array}{cc}{cos}\left({\uptheta\:}/2\right)&\:{sin}\left({\uptheta\:}/2\right)\\\:-{sin}\left({\uptheta\:}/2\right)&\:{cos}\left({\uptheta\:}/2\right)\end{array}\right),\:\mathrm{a}\mathrm{n}\mathrm{d}\:{S}_{z}\left(\theta\:\right)=\left(\begin{array}{cc}{e}^{i\left({\uptheta\:}/2\right)}&\:0\\\:0&\:{e}^{-i\left({\uptheta\:}/2\right)}\end{array}\right).$$

These matrices are unitary $\:\left({\mathrm{S}}_{i}{S}_{i}^{{\dagger} }=I\right)$ and therefore invertible, making them suitable for reversible encryption operations.

Within the framework, quantum spin matrices are employed as lightweight confusion and diffusion primitives rather than as sources of cryptographic hardness. The matrices and their quantized forms are fully determined by the public phase $\:\theta\:$, while the selection schedule alone is key-driven (Sect. 3.2), a distinction analyzed in Sect. 4.1. The role of the spin matrices is threefold:

1. *Confusion*: Spin-matrix selection is keyed and pseudorandom, obscuring the relationship between plaintext pixels and ciphertext values.

2) *Diffusion*: Matrix-based transformations propagate small changes across image blocks, enhancing avalanche behavior.

3) *Reversibility*: The deterministic, schedule-aligned application of identical quantized matrices guarantees lossless decryption when applied in reverse order.

Because XOR operates on integer operands while spin-matrix entries are complex, each entry * z=a+bi* of a selected matrix * Q* is mapped to an 8-bit integer through a deterministic magnitude-based quantization function $\:q:\mathbb{C}\to\:\left\{0,\dots\:,\:255\right\}$, defined as $\:q\left(z\right)=uint8\left(\lfloor\:s\left|z\right|\rceil\right)$, where $\:\left|z\right|=\sqrt{{a}^{2}+{b}^{2}}$ is the magnitude, $\:\lfloor\cdot\:\rceil$ denotes rounding to the nearest integer, and $\:uint8\left(\cdot\:\right)$ saturates the result to the range $\:\left[0,\:255\right]$. Since the spin matrices are unitary, all entry magnitudes lie in $\:\left[0,\:1\right]$, and the public scaling constant * s=255* maps this range onto the full 8-bit domain. The phase parameter $\:\theta\:$ is a public, shared value; consequently, both sender and receiver construct identical matrices * Q* and identical quantized representations $\:q\left(Q\right)$. For an image block * B*, the transformation is therefore $\:B{\prime\:}=q\left(Q\right)\oplus\:B$.

The mapping * q* is many-to-one (entries sharing a magnitude map to the same byte) and is therefore not invertible in isolation. Reversibility of the scrambling layer does not, however, require inverting * q*. Because XOR is involutive and the identical quantized matrix $\:q\left(Q\right)$ is applied during both encryption and decryption, being deterministically reconstructed from the public phase $\:\theta\:$ and the key-driven schedule, recovery is exact at the byte level: $\:q\left(Q\right)\oplus\left(q\left(Q\right)\oplus\:B\right)=B$. No floating-point inversion of the complex matrices is performed, so the finite-precision mapping introduces no decryption error. Correctness depends only on byte-level XOR equivalence, not on the numerical precision or injectivity of * q*.

The magnitude-based quantization induces a bounded representational loss relative to the exact complex entries, which affects only the statistical diffusion behavior of the scrambling layer and is evaluated empirically in Sect. 4.3 (entropy, NPCR, UACI), it does not affect correctness. Because the scrambling layer is public and unkeyed at the level of cryptographic hardness (any party knowing θ can reconstruct $\:q\left(Q\right)$) the mapping exposes no secret material and introduces no algebraic attack surface against the cryptographic core, whose security rests entirely on the sntrup761 KEM and the authenticated encryption layer. No standalone cryptographic security claims are made for this transformation.

## Methodology

This section presents the proposed post-quantum image encryption methodology. The scheme integrates lattice-based KEM, key derivation, spin-matrix-based confusion and diffusion, and authenticated encryption into a unified framework. The design explicitly separates cryptographic hardness from lightweight transformation layers, ensuring that post-quantum security relies on well-established assumptions while maintaining computational efficiency. Section 3.1 introduces the system model and threat assumptions and provides a high-level overview of the encryption and decryption workflow. Section 3.2 details the encryption procedure and presents the corresponding pseudocode. Section 3.3 describes the decryption process, including authentication verification and inverse transformations.

### System overview and threat model

The proposed scheme is a post-quantum image encryption framework designed for secure transmission over an untrusted channel. The system consists of two legitimate entities, such as a sender, who encrypts an image, and a receiver, who decrypts it. An adversary is assumed to have full control over the communication channel.

The encryption process operates on an input image $\:M\in\:{\left\{0,\:1\right\}}^{8\times\:H\times\:W\times\:C}$, where $\:H,\:W,\:\mathrm{a}\mathrm{n}\mathrm{d}\:C$ denote the height, width, and number of channels, respectively. The system integrates four cryptographic components:

- A post-quantum KEM based on sntrup761,
- A Key Derivation Function (HKDF) for role-separated key generation,
- A spin-matrix-based scrambling layer driven by a nondeterministic finite automaton (NDFA),
- An Authenticated Encryption with Associated Data scheme.

**Fig. 2:**
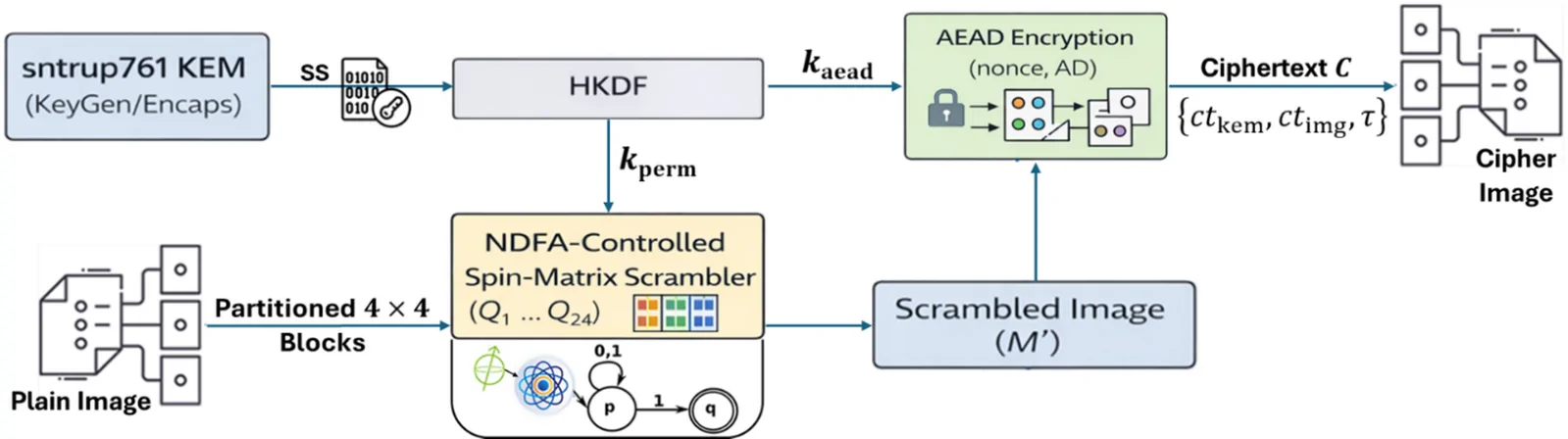
Proposed image encryption model.

A complete operational flowchart of the framework, including the authentication-abort branch, is provided in Fig. 2.

## Data flow

The encryption operation can be viewed as a composition of four transformations, such as $\:\mathcal{E}={\mathcal{E}}_{\mathrm{A}\mathrm{E}\mathrm{A}\mathrm{D}}\circ\:{\mathcal{S}}_{\mathrm{s}\mathrm{p}\mathrm{i}\mathrm{n}}\circ\:{\mathcal{K}}_{\mathrm{H}\mathrm{K}\mathrm{D}\mathrm{F}}\circ\:{\mathcal{K}}_{\mathrm{K}\mathrm{E}\mathrm{M}}$, where each stage has a clearly separated function:

- *Post-quantum key establishment*: A shared secret is established using sntrup761 KEM as.

5 $$\:\left({ct}_{\mathrm{k}\mathrm{e}\mathrm{m}},\:ss\right)\leftarrow\:\mathrm{s}\mathrm{n}\mathrm{t}\mathrm{r}\mathrm{u}\mathrm{p}761.\mathrm{E}\mathrm{n}\mathrm{c}\mathrm{a}\mathrm{p}\mathrm{s}\left({p}_{k}\right).$$

- *Key derivation*: The shared secret is expanded into independent cryptographic keys as.

6 $$\:\left({k}_{\mathrm{p}\mathrm{e}\mathrm{r}\mathrm{m}},\:{k}_{\mathrm{a}\mathrm{e}\mathrm{a}\mathrm{d}}\right)\leftarrow\:\mathrm{H}\mathrm{K}\mathrm{D}\mathrm{F}\left(\mathrm{s}\mathrm{s}\parallel\mathrm{c}\mathrm{t}\mathrm{x}\right).$$

- *Spin-matrix-based scrambling*: Using permutation key, plaintext image is transformed into a scrambled image as.

7 $$\:{M}^{{\prime\:}}=\mathrm{S}\mathrm{c}\mathrm{r}\mathrm{a}\mathrm{m}\mathrm{b}\mathrm{l}\mathrm{e}\left(M;\:{k}_{\mathrm{p}\mathrm{e}\mathrm{r}\mathrm{m}},\:\theta\:\right).$$

- *Authenticated encryption*: The scrambled image is encrypted and authenticated as.

8 $$\:\left({ct}_{\mathrm{i}\mathrm{m}\mathrm{g}},\:\tau\:\right)\leftarrow\:{\mathrm{A}\mathrm{E}\mathrm{A}\mathrm{D}}_{\mathrm{E}\mathrm{n}\mathrm{c}\mathrm{r}\mathrm{y}\mathrm{p}\mathrm{t}}\left({k}_{\mathrm{a}\mathrm{e}\mathrm{a}\mathrm{d}},\:\mathrm{n}\mathrm{o}\mathrm{n}\mathrm{c}\mathrm{e},\:{M}^{{\prime\:}},\:\mathrm{A}\mathrm{D}\right).$$

The final transmitted ciphertext is:

9 $$\:C=\left\{{ct}_{\mathrm{k}\mathrm{e}\mathrm{m}},\:\mathrm{n}\mathrm{o}\mathrm{n}\mathrm{c}\mathrm{e},\:\mathrm{A}\mathrm{D},\:{ct}_{\mathrm{i}\mathrm{m}\mathrm{g}},\:\tau\:\right\}.$$

All algorithmic details of these transformations are specified in Sect. 3.2 and 3.3.

## Threat model and security scope

The adversary is assumed to have the following capabilities: (1) Full control of the communication channel, including interception, replay, modification, and injection of messages; (2) Access to encryption oracles (chosen-plaintext attacks–CPA); (3) Adaptive access to decapsulation and decryption oracles (chosen-ciphertext attacks–CCA); and (4) Polynomial-time classical and quantum computational resources. The adversary does not possess the receiver’s private key or the derived session keys. Under this threat model, the proposed scheme aims to achieve:

- Post-quantum key security, relying on the IND-CCA security of sntrup761,
- Confidentiality of image data, provided by AEAD (IND-CPA),
- Integrity and authenticity, enforced by AEAD tag verification, and.
- Layered security separation, where spin matrices and NDFA scheduling are assumed public and do not contribute cryptographic hardness.

The analysis addresses adversaries operating at the protocol level. Physical side-channel leakage, including timing, power, and cache behavior of the implementation, is outside the present scope and is treated as an implementation-hardening concern; constant-time decapsulation is assumed for sntrup761, consistent with its reference implementation. Although the NDFA schedule is deterministic given $\:{k}_{\mathrm{p}\mathrm{e}\mathrm{r}\mathrm{m}}$, on the decryption path it is never applied to unauthenticated data. AEAD verification precedes any inverse scrambling (Sect. 3.3), so an adversary cannot induce schedule-dependent behavior on forged ciphertext. The ability of an adversary to influence $\:{k}_{\mathrm{p}\mathrm{e}\mathrm{r}\mathrm{m}}$ indirectly is analyzed in Sect. 4.1.

### Encryption procedure

**Fig. 3:**
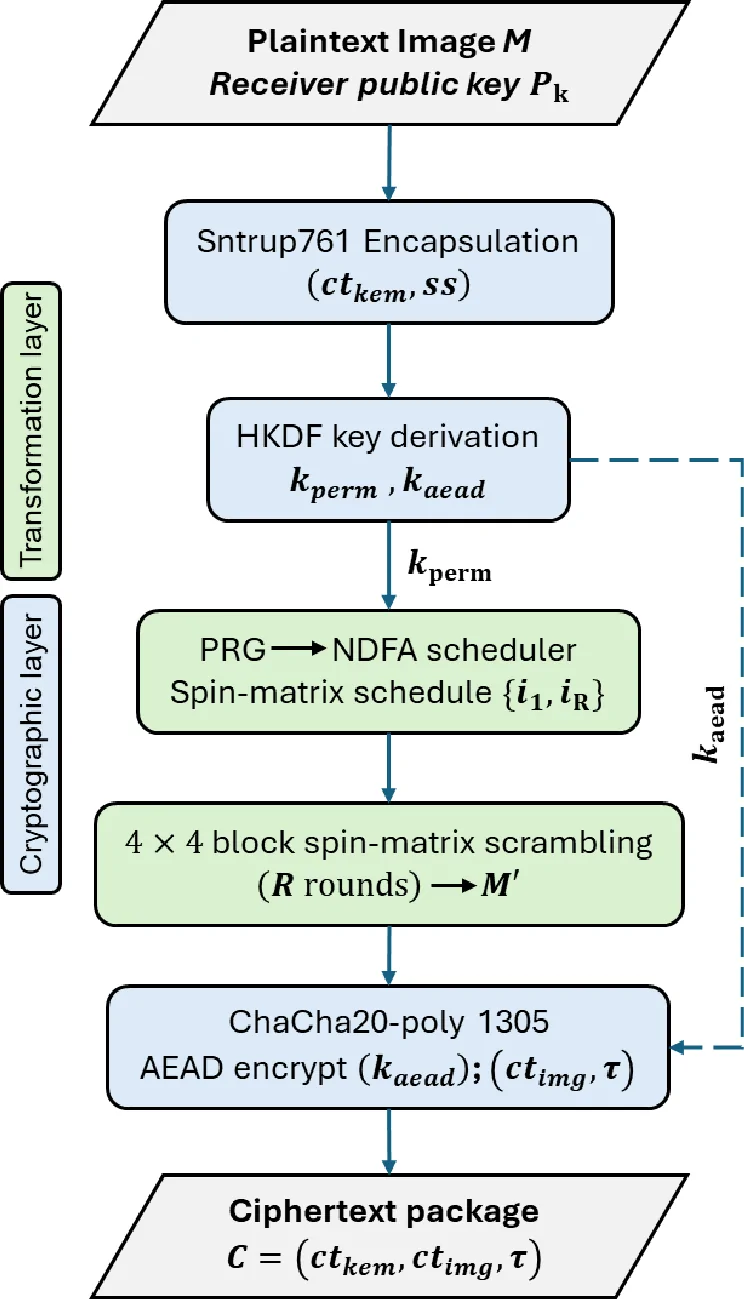
Proposed image encryption model.

This subsection presents the encryption procedure with algorithmic specification (Algorithm 1) and a flowchart (Fig. 3) corresponding to the data flow executed by the sender. Given an input image * M* and the receiver’s public key $\:{p}_{k}$, encryption proceeds as follows:

**Step 1 –** *Post-Quantum Key Encapsulation*: The sender establishes a shared secret using sntrup761 as $\:\left({ct}_{\mathrm{k}\mathrm{e}\mathrm{m}},\:ss\right)\leftarrow\:\mathrm{s}\mathrm{n}\mathrm{t}\mathrm{r}\mathrm{u}\mathrm{p}761.\mathrm{E}\mathrm{n}\mathrm{c}\mathrm{a}\mathrm{p}\mathrm{s}\left({p}_{k}\right)$.

**Step 2 –** *Key Derivation*: The shared secret is expanded using HKDF as $\:\left({k}_{\mathrm{p}\mathrm{e}\mathrm{r}\mathrm{m}},\:{k}_{\mathrm{a}\mathrm{e}\mathrm{a}\mathrm{d}}\right)\leftarrow\:\mathrm{H}\mathrm{K}\mathrm{D}\mathrm{F}\left(\mathrm{s}\mathrm{s}\parallel\mathrm{c}\mathrm{t}\mathrm{x}\right),$ where $\:\mathrm{c}\mathrm{t}\mathrm{x}$ encodes public parameters such as image dimensions, block size, number of rounds, phase angle, and protocol identifiers.

**Step 3 –** *Spin-Matrix Set and NDFA Scheduling*: A fixed set of 24 invertible matrices $\:\mathcal{Q}=\left\{{Q}_{1},\:{Q}_{2},\:\dots\:,\:{Q}_{24}\right\}$, $\:{Q}_{i}\in\:{\mathbb{C}}^{4\times\:4}$, is constructed from the spin-rotation operators of Sect. 2.2. Each $\:{Q}_{i}$ is formed as a $\:2\times\:2$ block matrix whose four $\:2\times\:2$ blocks are a distinct permutation of the operator set $\:\left\{I,\:{S}_{x}\left(\theta\:\right),\:{S}_{y}\left(\theta\:\right),\:{S}_{z}\left(\theta\:\right)\right\}$, where * I* is the identity and $\:{S}_{x}$, $\:{S}_{y}$, $\:{S}_{z}$ are the spin-rotation operators parameterized by the public phase $\:\theta\:$. The * 4!=24* permutations yield the 24 distinct members of * Q*, and every entry has magnitude in $\:\left[0,\:1\right]$, consistent with the quantization map * q* of Sect. 2.2. The set is fixed and public; only the order in which its members are applied is secret. The scheduler is a nondeterministic finite automaton $\:A=\left(S,\:{\Sigma\:},\:\delta\:,\:{s}_{0},\:\pi\:\right)$, where * S* is a finite set of internal states, $\:{\Sigma\:}$ is the input alphabet of byte symbols emitted by the pseudorandom generator, $\:\delta\::S\times\:{\Sigma\:}\to\:{2}^{S}$ is the nondeterministic transition relation mapping each (state, symbol) pair to a set of admissible successor states, $\:{s}_{0}\epsilon{S}$ is the initial state seeded deterministically from the permutation key $\:{k}_{\mathrm{p}\mathrm{e}\mathrm{r}\mathrm{m}}$, and $\:\pi\::S\to\:\left\{1,\:2,\:\dots\:,\:24\right\}$ is an output map returning the index of the selected spin matrix. A keyed pseudorandom sequence $\:{v}_{1},\:{v}_{2},\dots\:,\:{v}_{R}\leftarrow\:\mathrm{P}\mathrm{R}\mathrm{G}\left({k}_{\mathrm{p}\mathrm{e}\mathrm{r}\mathrm{m}}\right)$ is generated, where * R* is the number of scrambling rounds. At round * r*, the symbol $\:{v}_{r}$ resolves the nondeterminism of $\:\delta\:$: among the admissible successors $\:\delta\:\left({s}_{{r}^{-1}},\:{v}_{r}\right)$, the symbol selects a unique successor state $\:{s}_{r}$, yielding the output index $\:{i}_{r}=\pi\:\left({s}_{r}\right)$. The automaton is therefore structurally nondeterministic (multiple transitions are admissible from each state) yet a given $\:{k}_{\mathrm{p}\mathrm{e}\mathrm{r}\mathrm{m}}$ resolves it to a single, reproducible run. The same $\:{k}_{\mathrm{p}\mathrm{e}\mathrm{r}\mathrm{m}}$ at the receiver regenerates the identical schedule $\:\left\{{i}_{1},\:{i}_{2},\dots\:,\:{i}_{R}\right\}$, which is what makes decryption deterministic. Without $\:{k}_{\mathrm{p}\mathrm{e}\mathrm{r}\mathrm{m}}$ the realized path is one of exponentially many admissible schedules, which provides the confusion contribution of the layer. To ensure each matrix is selected with probability * 1/24* and to avoid the bias that arises when a uniform byte $\:\left(0-255\right)$ is reduced modulo * 24*, the index is drawn by rejection sampling. A $\:\mathrm{P}\mathrm{R}\mathrm{G}$ byte * b* is accepted only if * b<240* (the largest multiple of * 24* below * 256*), giving $\:{i}_{r}=\left(b\:mod\:24\right)+1$; otherwise, * b* is discarded and the next byte is drawn. This guarantees an exactly uniform distribution over the * 24* matrices, so no matrix is systematically favored across the * R* rounds.

**Step 4 –** *Image Scrambling*: The image is partitioned into non-overlapping $\:4\times\:4$ blocks. For each scrambling round and each block, a quantized XOR operation with the selected spin matrix is applied. After all rounds, the scrambled image $\:{M}^{{\prime\:}}$ is obtained.

**Step 5 –** *Authenticated Encryption*: The scrambled image is encrypted using an AEAD scheme as $\:\left({ct}_{\mathrm{i}\mathrm{m}\mathrm{g}},\:\tau\:\right)\leftarrow\:{\mathrm{A}\mathrm{E}\mathrm{A}\mathrm{D}}_{\mathrm{E}\mathrm{n}\mathrm{c}\mathrm{r}\mathrm{y}\mathrm{p}\mathrm{t}}\left({k}_{\mathrm{a}\mathrm{e}\mathrm{a}\mathrm{d}},\:\mathrm{n}\mathrm{o}\mathrm{n}\mathrm{c}\mathrm{e},\:{M}^{{\prime\:}},\:\mathrm{A}\mathrm{D}\right)$, where $\:\tau\:$ is the authentication tag and AD includes public metadata.

The scheme has four public parameters: the block size $\:\left(4\times\:4\right)$, the matrix-set size $\:\left(24\right)$, the rotation phase $\:\theta\:$, and the number of rounds * R*. The block size and set size follow from the $\:4\times\:4$ block structure of the spin matrices. The influence of * R* on diffusion strength and computational cost is examined empirically in Sect. 4.3, where * R=4* is selected as the point beyond which the diffusion metrics saturate.

### Decryption procedure

**Fig. 4:**
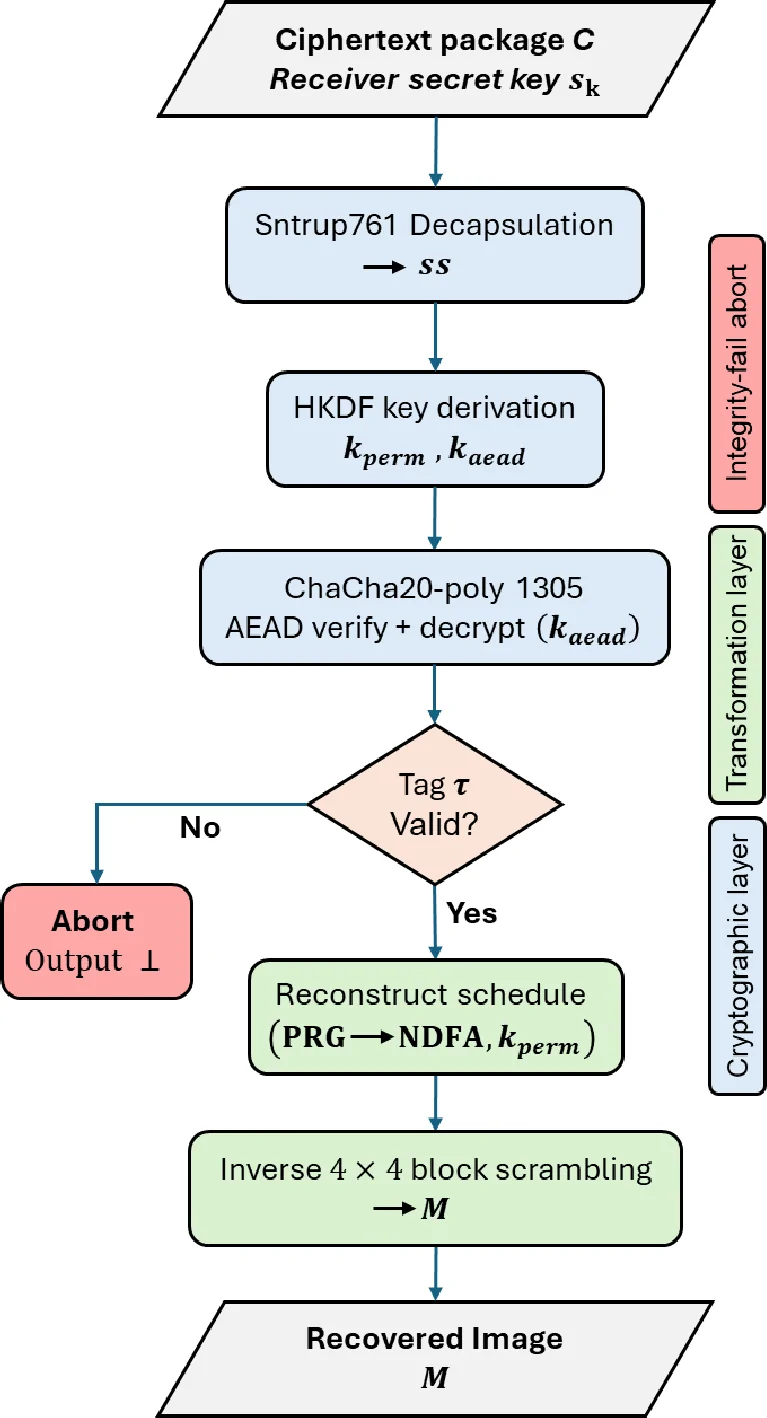
Flowchart of decryption procedure (Algorithm 2).

This subsection describes the decryption process executed by the receiver to recover the original image from the received ciphertext. Figure 4 presents the flowchart of the decryption process. The decryption procedure reverses the encryption steps described in Sect. 3.2 and enforces authentication prior to any image recovery. The complete procedure is summarized in Algorithm 2.

Let the received ciphertext package be $\:C=\left\{{ct}_{\mathrm{k}\mathrm{e}\mathrm{m}},\:\mathrm{n}\mathrm{o}\mathrm{n}\mathrm{c}\mathrm{e},\:\mathrm{A}\mathrm{D},\:{ct}_{\mathrm{i}\mathrm{m}\mathrm{g}},\:\tau\:\right\}$. The receiver also possesses the private key $\:{s}_{k}$ generated during the sntrup761 key generation phase.

**Step 1 –** *Post-Quantum Key Decapsulation*: The receiver recovers the shared secret by decapsulating the KEM ciphertext as $\:ss\leftarrow\:\mathrm{s}\mathrm{n}\mathrm{t}\mathrm{r}\mathrm{u}\mathrm{p}761.\mathrm{D}\mathrm{e}\mathrm{c}\mathrm{a}\mathrm{p}\mathrm{s}\left({s}_{k},\:{ct}_{\mathrm{k}\mathrm{e}\mathrm{m}}\right)$. Due to the IND-CCA security of sntrup761, this operation either recovers the correct shared secret or returns a pseudorandom value indistinguishable from random.

**Step 2 –** *Key Derivation*: Using the recovered shared secret, the receiver derives the same role-separated keys as the sender $\:\left({k}_{\mathrm{p}\mathrm{e}\mathrm{r}\mathrm{m}},\:{k}_{\mathrm{a}\mathrm{e}\mathrm{a}\mathrm{d}}\right)\leftarrow\:\mathrm{H}\mathrm{K}\mathrm{D}\mathrm{F}\left(\mathrm{s}\mathrm{s}\parallel\mathrm{c}\mathrm{t}\mathrm{x}\right),$ where the context $\:\mathrm{c}\mathrm{t}\mathrm{x}$ is reconstructed from public parameters contained in the associated data $\:\mathrm{A}\mathrm{D}$.

**Step 3 –** *Authenticated Decryption*: Before any scrambling reversal, the receiver performs authenticated decryption as $\:{M}^{{\prime\:}}\leftarrow\:{\mathrm{A}\mathrm{E}\mathrm{A}\mathrm{D}}_{\mathrm{D}\mathrm{e}\mathrm{c}\mathrm{r}\mathrm{y}\mathrm{p}\mathrm{t}}\left({k}_{\mathrm{a}\mathrm{e}\mathrm{a}\mathrm{d}},\:\mathrm{n}\mathrm{o}\mathrm{n}\mathrm{c}\mathrm{e},\:{ct}_{\mathrm{i}\mathrm{m}\mathrm{g}},\:\mathrm{A}\mathrm{D},\:\tau\:\right)$. If authentication fails, the decryption process is immediately aborted, and the output is set to $\:\perp\:$. This step prevents replay, forgery, and adaptive CCA on the image data.

**Step 4 –** *Spin-Matrix Scheduling Reconstruction*: If authentication succeeds, the receiver reconstructs the spin-matrix scheduling process. Using the permutation key $\:{k}_{\mathrm{p}\mathrm{e}\mathrm{r}\mathrm{m}}$, the same pseudorandom sequence is generated as $\:\left({v}_{1},\:{v}_{2},\:\dots\:,\:{v}_{\mathrm{R}}\right)\leftarrow\:\mathrm{P}\mathrm{R}\mathrm{G}\left({k}_{\mathrm{p}\mathrm{e}\mathrm{r}\mathrm{m}}\right)$. The nondeterministic finite automaton processes this sequence to reproduce the same index sequence, $\:{i}_{r}=\mathrm{N}\mathrm{D}\mathrm{F}\mathrm{A}\left({v}_{r}\right),\:\:\:\:r=1,\:2,\:\dots\:,\:R$. The set of spin matrices $\:\mathcal{Q}=\left\{{Q}_{1},\:{Q}_{2},\:\dots\:,\:{Q}_{24}\right\}$ is reconstructed using the shared phase parameter $\:\theta\:$.

**Step 5 –** *Inverse Image Descrambling*: The decrypted scrambled image $\:{M}^{{\prime\:}}$ is partitioned into non-overlapping $\:4\times\:4$ blocks as $\:{M}^{{\prime\:}}=\left\{{B}_{1}^{\left(R\right)},\:{B}_{2}^{\left(R\right)},\:\dots\:,\:{B}_{N}^{\left(R\right)}\right\}$. Since * XOR* is an involutive operation, the inverse scrambling is performed by applying the same transformations in reverse order as $\:{B}_{j}^{\left(r-1\right)}={Q}_{{i}_{r}}\oplus\:{B}_{j}^{\left(r\right)},\:\:\:\:r=R,\:R-1,\:\dots\:,\:1.$.

After all rounds are reversed, the original image blocks $\:{B}_{j}^{\left(0\right)}$ are obtained and reassembled to recover the plaintext image * M*.

**Figure:**
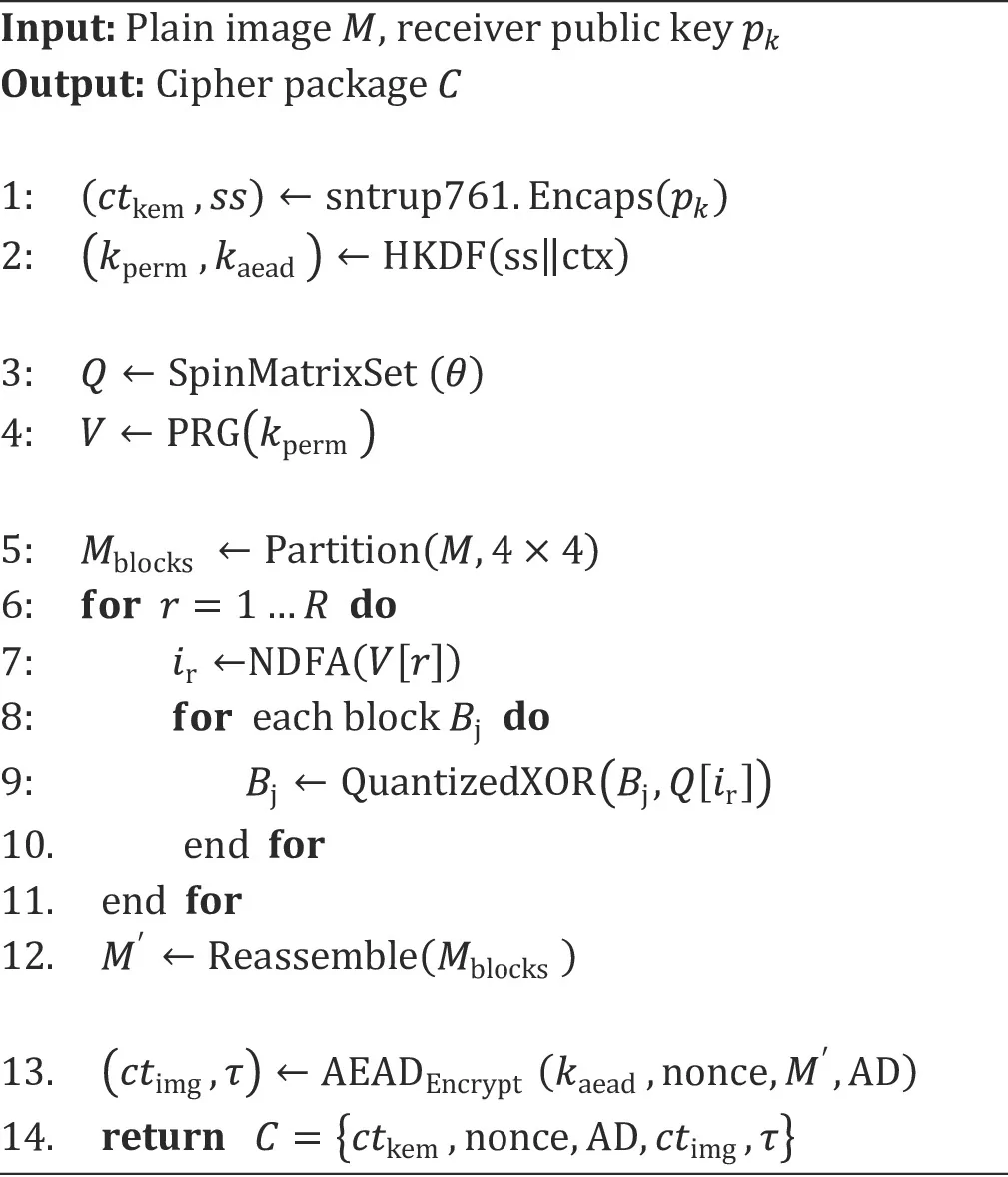
**Algorithm 1.** Post-Quantum Spin-NDFA Image Encryption

### Experiment

**Fig. 5:**
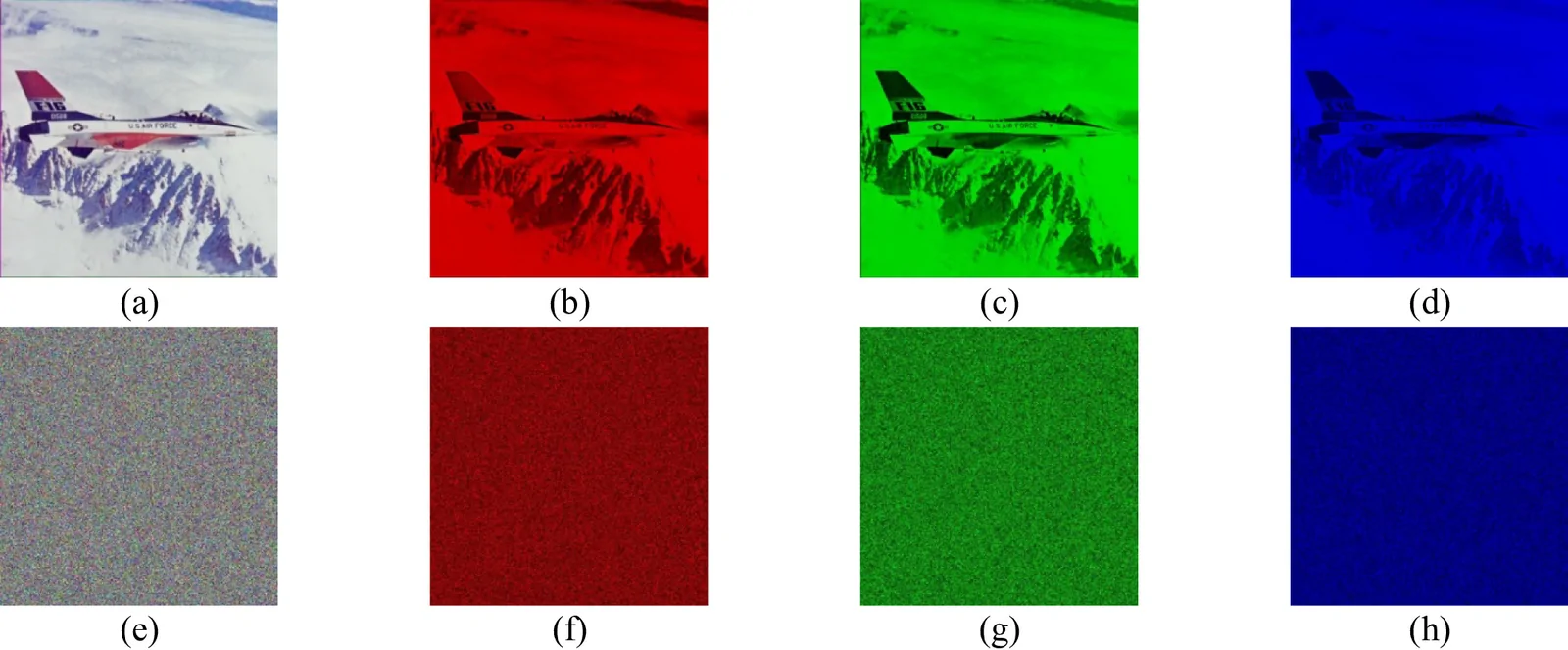
Layer wise encryption of airplane image. (a) Source image; (b-d) Extracted RGB layers; (e) Concatenated encrypted RGB layers; (f-h) Encrypted RGB layers.

All experiments were implemented and evaluated in MATLAB R2024a on a 64-bit Windows 11 workstation with a 13th-generation Intel Core i9-13900HX processor (24 cores/32 threads; P-core base 2.20 GHz, up to 5.40 GHz; E-core base 1.60 GHz, up to 3.90 GHz) and 32 GB of RAM. The lattice-based key establishment used the sntrup761 parameter set, HKDF performed role-separated key derivation, and authenticated encryption was implemented using ChaCha20-Poly1305. Evaluation used several standard images from the USC-SIPI database^37^ spanning varied texture and structure, including Airplane, Baboon, Pepper, House, and Boat. Each image was processed at $\:256\times\:256,\:512\times\:512,\:\mathrm{a}\mathrm{n}\mathrm{d}\:1024\times\:1024$ to characterize scalability. Layer-wise encryption results for the Airplane image are shown in Fig. 5.

Encryption and decryption latency were measured using MATLAB’s timeit function, which averages over repeated executions after warm-up; reported values are the mean of * k=20* independent runs. Peak working-set memory was recorded through the memory function as the increase in MemUsedMATLAB across each operation. Per-stage timings were additionally recorded for key encapsulation, key derivation, spin-matrix scrambling, and authenticated encryption. Table 2 reports runtime and memory across resolutions, and Table 3 the per-stage breakdown at $\:512\times\:512$.

The measured encryption and decryption times scale approximately linearly with pixel count, while key encapsulation and derivation remain effectively constant across image sizes, empirically confirming the $\:\mathcal{O}\left(\mathrm{H}\times\:\mathrm{W}\times\:\mathrm{C}\right)$ complexity established analytically in Sect. 4.2.

**Figure:**
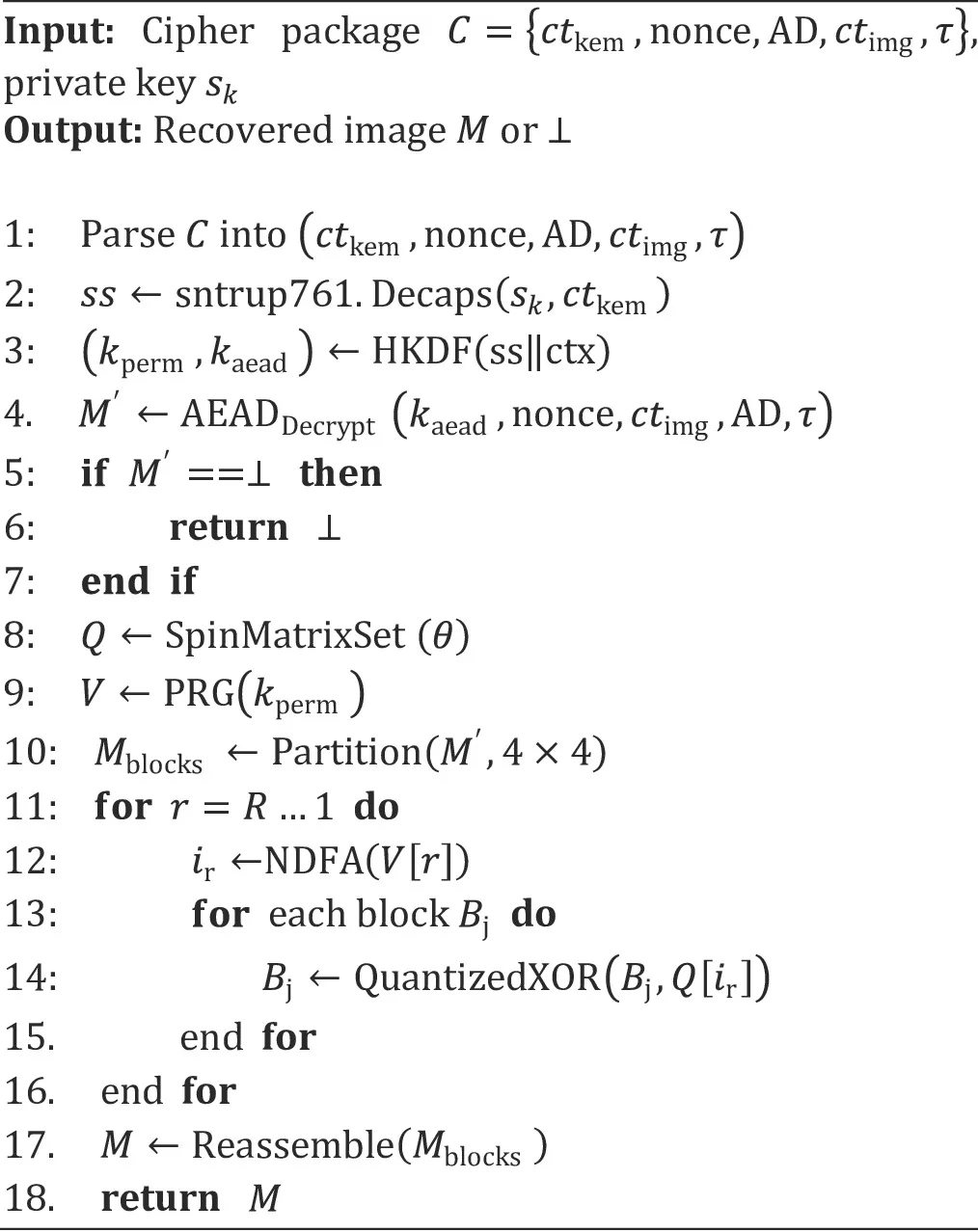
**Algorithm 2.** Post-Quantum Spin-NDFA Image Decryption

**Table 2:** Measured encryption/decryption time and peak memory across image resolutions.

Image size	Pixels	Enc. time (ms)	Dec. time (ms)	Peak memory (MB)
$\:256\times\:256$	65,536	14.2	13.6	18.4
$\:512\times\:512$	262,144	56.1	54.3	42.7
$\:1024\times\:1024$	1,048,576	223.8	216.5	138.5

**Table 3:** Per-stage encryption cost at $\:512\times\:512$.

Stage	Time (ms)	% of total	Scales with image
sntrup761 encapsulation	0.82	1.5%	No (fixed/session)
HKDF derivation	0.11	0.2%	No (fixed)
NDFA spin-matrix scrambling	30.8	54.9%	Yes (linear)
ChaCha20-Poly1305 AEAD	24.4	43.4%	Yes (linear)
**Total**	**56.1**	**100%**	**–**

## Security and performance analysis

This section provides a comprehensive evaluation of the proposed framework from both cryptographic and image-processing perspectives. The analysis is structured to clearly separate formal security guarantees derived from standardized cryptographic primitives from statistical and empirical evaluations of the image transformation layer. First, we analyze the cryptographic properties of the scheme, including key establishment security, confidentiality, integrity, and resistance to adaptive adversaries. We then evaluate computational complexity and communication overhead to assess practical efficiency. Finally, statistical, robustness, and comparative analyses are presented to demonstrate the effectiveness of the spin-matrix scrambling layer in enhancing diffusion and maintaining stable behavior under perturbations.

### Cryptographic security analysis

This subsection analyzes the security properties of the proposed framework under the threat model defined in Sect. 3. Unlike conventional image encryption schemes that rely primarily on statistical evaluation, the security of the proposed method is grounded in formally analyzed cryptographic primitives. Specifically, security relies on the IND-CCA guarantees of the lattice-based sntrup761 key encapsulation mechanism and the confidentiality and integrity properties of authenticated encryption. The following analysis clarifies how these guarantees collectively provide resistance against classical and quantum-capable adversaries.

## Security goals

The proposed framework is designed to achieve cryptographic security against both classical and quantum-capable adversaries while maintaining practical suitability for image data transmission. The primary security objectives are defined as follows:

- Post-quantum secure key establishment, ensuring that session keys remain secure against adversaries equipped with polynomial-time quantum computation.
- Confidentiality of image data, preventing adversaries from distinguishing encrypted images from random data under CPA.
- Integrity and authenticity, ensuring that any modification or forgery of transmitted ciphertext is detected prior to image recovery.
- Resistance to adaptive CCA, preventing adversaries from exploiting decryption behavior to obtain information about secret keys or plaintext images.

These goals are realized through a layered architecture in which cryptographic security is provided by standardized primitives, while image-specific transformations enhance diffusion without introducing additional hardness assumptions.

## Key establishment security

The proposed scheme employs the lattice-based sntrup761 KEM to establish a shared secret between communicating parties. The security of sntrup761 is based on hard lattice problems and is designed to remain secure against both classical and quantum adversaries.

During encryption, the sender generates a shared secret through encapsulation, while the receiver reconstructs the same secret via decapsulation. The underlying KEM provides IND-CCA security, implying that an adversary cannot distinguish the derived shared secret from random even when granted adaptive access to decapsulation oracles. Consequently, any attempt to manipulate or replace the encapsulated ciphertext does not reveal useful information about the session key.

The shared secret produced by the KEM is subsequently expanded using HKDF into role-separated keys. This derivation ensures key independence between the scrambling process and authenticated encryption stage, preventing cross-layer key leakage or misuse.

## Confidentiality and integrity guarantees

After image scrambling, confidentiality and authenticity are enforced through AEAD construction. The AEAD layer provides two essential guarantees:

- *Confidentiality (IND-CPA security)*: Encrypted image data remains computationally indistinguishable from random ciphertext.
- *Integrity and authenticity (INT-CTXT security)*: Any ciphertext modification, insertion, or forgery is detected during decryption.

A critical design property of the proposed framework is that authentication verification occurs before inverse scrambling operations. If the authentication tag verification fails, the decryption procedure terminates and outputs ⊥, preventing adversaries from observing partial image recovery or exploiting structural properties of the scrambling layer. Therefore, the overall confidentiality and integrity of the proposed system rely directly on the security guarantees of the underlying AEAD primitive rather than on statistical properties of the image transformations.

Because the AEAD layer provides IND-CPA security over the scrambled image under a fresh key $\:{k}_{\mathrm{a}\mathrm{e}\mathrm{a}\mathrm{d}}$ and nonce, the framework resists CPA. An adversary able to obtain encryptions of chosen images cannot distinguish ciphertexts or recover key material. Known-plaintext attacks (KPA), which are strictly weaker than CPA, are therefore also resisted. This guarantee derives from the authenticated-encryption layer and is independent of the scrambling stage; as a complementary, defense-in-depth indicator at the transformation layer, the plaintext-sensitivity metrics NPCR and UACI reported in Sect. 4.3 confirm that a single-pixel plaintext change induces near-ideal diffusion across the scrambled image.

## Resistance to adaptive attacks

The threat model assumes an adversary capable of intercepting, modifying, replaying, and injecting ciphertexts, as well as performing adaptive chosen-plaintext and chosen-ciphertext attacks. The proposed framework mitigates such attacks through the joint security properties of the KEM and AEAD layers.

First, the IND-CCA security of the sntrup761 KEM prevents adversaries from deriving meaningful information from manipulated encapsulation ciphertexts. Second, AEAD authentication ensures that malicious modifications of encrypted image data are rejected prior to decryption. As a result, adaptive probing attempts do not leak information about plaintext images or secret keys.

The NDFA-driven spin-matrix scrambling layer is intentionally treated as a deterministic transformation controlled by derived keys and does not constitute an independent security assumption. Instead, it serves to enhance spatial diffusion and reduce structural correlations in image data prior to authenticated encryption. Hence, the overall security of the framework reduces to the combined guarantees of the post-quantum KEM and AEAD primitives.

Two further adversarial scenarios identified in the threat model require explicit treatment. First, although the NDFA schedule is deterministic, it cannot be manipulated indirectly through $\:{k}_{\mathrm{p}\mathrm{e}\mathrm{r}\mathrm{m}}$. The permutation key is derived from the KEM shared secret through HKDF, and the IND-CCA security of sntrup761 ensures that the shared secret is indistinguishable from random under adaptive decapsulation queries. An adversary therefore has no means of biasing or controlling $\:{k}_{\mathrm{p}\mathrm{e}\mathrm{r}\mathrm{m}}$, and any tampering with the transmitted ciphertext is detected by AEAD verification before the schedule is ever reconstructed (Sect. 3.3); the scrambling layer is never applied to unauthenticated data. Second, physical side-channel leakage, such as timing, power, or cache behavior, is outside the protocol-level threat model (Sect. 3.1). Constant-time implementation of decapsulation and authenticated decryption is assumed, consistent with the sntrup761 reference implementation, and is recommended as a deployment-hardening measure.

## Composite security argument

The guarantees described above apply to individual primitives. The following argument establishes that their composition preserves these properties under the threat model of Sect. 3. The encryption pipeline is a functional composition $\:\mathcal{E}={\mathcal{E}}_{\mathrm{A}\mathrm{E}\mathrm{A}\mathrm{D}}\circ\:{\mathcal{S}}_{\mathrm{s}\mathrm{p}\mathrm{i}\mathrm{n}}\circ\:{\mathcal{K}}_{\mathrm{H}\mathrm{K}\mathrm{D}\mathrm{F}}\circ\:{\mathcal{K}}_{\mathrm{K}\mathrm{E}\mathrm{M}}$ (Sect. 3.1). Confidentiality is analyzed through a sequence of transitions from the real scheme. First, by the IND-CCA security of sntrup761, the shared secret * ss* is replaced by a uniformly random value. Any adversary distinguishing this transition breaks the KEM. Second, by the pseudorandomness of HKDF, the derived pair ($\:{k}_{\mathrm{p}\mathrm{e}\mathrm{r}\mathrm{m}}$, $\:{k}_{\mathrm{a}\mathrm{e}\mathrm{a}\mathrm{d}}$) is replaced by independent uniform keys, so that the scrambling and encryption layers share no key material. Third, because the spin-matrix scrambling $\:{S}_{\mathrm{s}\mathrm{p}\mathrm{i}\mathrm{n}}$ is computable without knowledge of $\:{k}_{\mathrm{a}\mathrm{e}\mathrm{a}\mathrm{d}}$, any adversary achieving an IND-CPA advantage against the composition yields an adversary with the same advantage against the AEAD layer alone. The reduction simply applies $\:{S}_{\mathrm{s}\mathrm{p}\mathrm{i}\mathrm{n}}$ to the challenge plaintexts before forwarding them to the AEAD challenger. The confidentiality of the composition therefore reduces to the IND-CPA and INT-CTXT security of the AEAD layer keyed by $\:{k}_{\mathrm{a}\mathrm{e}\mathrm{a}\mathrm{d}}$, with the KEM and HKDF transitions contributing only negligible advantage. No independent security notion is claimed for the scrambling layer.

This reduction resolves the role of the scrambling layer directly. The spin matrices and their quantized forms are fully public, being determined by the phase $\:\theta\:$, while only the selection schedule is key-derived from $\:{k}_{perm}$. Since confidentiality reduces entirely to the AEAD layer, the secrecy of the schedule is not a cryptographic security boundary. An adversary who learned $\:{k}_{perm}$ would still face IND-CPA authenticated encryption, and conversely the confidentiality of the scheme does not depend on $\:{k}_{\mathrm{p}\mathrm{e}\mathrm{r}\mathrm{m}}$ remaining secret. The schedule is key-derived solely to make the diffusion session-specific and non-replayable, not to supply cryptographic hardness. Applying scrambling before AEAD thus introduces no distinguishing side channel. It is a deterministic preprocessing step whose output is fully protected by the subsequent authenticated-encryption layer.

The post-quantum security boundary can then be stated precisely. Quantum resistance derives solely from the sntrup761 KEM, whose IND-CCA security rests on hard lattice problems believed resistant to quantum attack. The scrambling and XOR operations contribute no independent quantum hardness, by design. Against Grover’s algorithm, the symmetric components retain at least half of their key strength in the exponent, so that the 256-bit AEAD key provides at least 128-bit post-quantum security. The scrambling schedule, even if subjected to Grover search, is not a confidentiality boundary as established above, and its reduced effective strength therefore does not weaken the scheme. The overall post-quantum security of the framework is consequently determined by the minimum of the sntrup761 KEM security and the 128-bit post-quantum strength of the authenticated-encryption layer, and the framework does not claim quantum hardness for the image-transformation layer.

### Complexity and computational analysis

This subsection evaluates the computational efficiency and scalability of the proposed framework. Since the design combines post-quantum key establishment with lightweight image transformation operations, it is important to quantify how each component contributes to overall complexity. The analysis decomposes the framework into key establishment, key derivation, scrambling operations, and authenticated encryption, allowing assessment of both computational cost and communication overhead with respect to image size.

## Computational cost

The computational complexity of the proposed framework can be analyzed by decomposing the encryption and decryption procedures into four primary components: post-quantum key encapsulation, key derivation, spin-matrix scrambling, and authenticated encryption.

- *Post-Quantum Key Encapsulation*: The sntrup761 KEM performs polynomial arithmetic over a fixed-degree ring (degree 761). Since the ring dimension and modulus are fixed by the parameter set, the computational cost of encapsulation and decapsulation is constant with respect to image size. Thus, the KEM contributes a fixed overhead per encryption session, independent of image resolution. For a single image transmission, the cost consists of one encapsulation operation at the sender and one decapsulation operation at the receiver. Because key establishment occurs once per session, its cost does not scale with the number of pixels.
- *Key Derivation*: HKDF operation expands the shared secret into independent keys. This process consists of hash-based extraction and expansion steps whose complexity is linear in the output key length. Since the derived keys have fixed size, the cost of HKDF is negligible compared to image processing operations and remains constant per session.
- *Spin-Matrix Scrambling*: Let the image be defined as $\:\mathrm{M}\in\:{\left\{0,\:1\right\}}^{8\times\:\mathrm{H}\times\:\mathrm{W}\times\:\mathrm{C}}$, where $\:\mathrm{H},\:\mathrm{W},\:\mathrm{a}\mathrm{n}\mathrm{d}\:\mathrm{C}$ denote height, width, and number of channels. The image is partitioned into non-overlapping $\:4\times\:4$ blocks. The total number of blocks is $\:\mathrm{N}=(\mathrm{H}\times\:\mathrm{W}\times\:\mathrm{C})/16$. For $\:\mathrm{R}$ scrambling rounds, each round applies a quantized XOR transformation to every block. Therefore, the scrambling complexity is $\:\mathcal{O}\left(\mathrm{R}\cdot\:\mathrm{N}\right)$. Since $\:\mathrm{R}$ is a small parameter determined by security configuration and independent of image size, the scrambling stage scales linearly with the number of pixels. Thus, the dominant cost of image transformation is linear in image resolution.
- *Authenticated Encryption*: The AEAD encryption stage processes the entire scrambled image once. Modern AEAD constructions (e.g., AES-GCM or ChaCha20-Poly1305) operate in streaming mode with linear complexity in the message length. Therefore, the AEAD complexity is $\:\mathcal{O}\left(\mathrm{H}\times\:\mathrm{W}\times\:\mathrm{C}\right)$. As a result, both encryption and decryption operations exhibit overall linear time complexity with respect to image size.
- ***Overall Complexity***: Combining all components, the total computational complexity for encrypting an image is $\:\mathcal{O}\left(\mathrm{R}\cdot\:\mathrm{N}\right)+\mathcal{O}\left(\mathrm{H}\mathrm{W}\mathrm{C}\right)+\mathcal{\:}\mathcal{O}\left(1\right)$. Since $\:\mathrm{N}\propto\:\mathrm{H}\mathrm{W}\mathrm{C}$, the overall complexity simplifies to $\:\mathcal{O}\left(\mathrm{H}\mathrm{W}\mathrm{C}\right)$. Therefore, the proposed framework scales linearly with image resolution, making it suitable for high-resolution image processing and real-time applications on desktop-class systems.

## Key size and ciphertext overhead

An important practical consideration is communication overhead introduced by post-quantum key establishment and authenticated encryption.

- *Key Material*: The sntrup761 parameter set has fixed public and private key sizes determined by its lattice dimension. Because keys are generated once and reused for multiple sessions if desired, they do not scale with image size. The session key derived via HKDF produces a permutation key $\:{\mathrm{k}}_{\mathrm{p}\mathrm{e}\mathrm{r}\mathrm{m}}$ and AEAD encryption key $\:{\mathrm{k}}_{\mathrm{a}\mathrm{e}\mathrm{a}\mathrm{d}}$. These keys have fixed length and do not increase with image resolution.
- *Ciphertext Structure*: The transmitted ciphertext package consists of $\:{\mathrm{c}\mathrm{t}}_{\mathrm{k}\mathrm{e}\mathrm{m}},\:\mathrm{n}\mathrm{o}\mathrm{n}\mathrm{c}\mathrm{e},\:\mathrm{A}\mathrm{D},\:{\mathrm{c}\mathrm{t}}_{\mathrm{i}\mathrm{m}\mathrm{g}},\:{\uptau\:}$. $\:{\mathrm{c}\mathrm{t}}_{\mathrm{k}\mathrm{e}\mathrm{m}}$ is the fixed-size KEM ciphertext, whereas $\:{\mathrm{c}\mathrm{t}}_{\mathrm{i}\mathrm{m}\mathrm{g}}$ is equal to the size of the original image (due to XOR-based scrambling and length-preserving AEAD). Thus, the only additional overhead beyond the original image size is one KEM ciphertext, one authentication tag, and a nonce with small associated metadata. Since these components are constant in size, the total overhead is $\:\left|\mathrm{C}\right|=\left|\mathrm{M}\right|+\mathcal{O}\left(1\right)$. Therefore, the communication overhead grows negligibly relative to image size and becomes proportionally insignificant for large images.

### Statistical evaluation of image encryption

The statistical evaluation presented in this subsection analyzes the diffusion and randomness characteristics of the encrypted images. While the cryptographic security of the proposed framework derives from the post-quantum KEM and AEAD primitives (Sect. 4.1), the following analyses assess whether the image transformation layer effectively disrupts spatial structures and statistical dependencies typically present in natural images. Experiments were conducted on standard benchmark images including Airplane, Baboon, Pepper, House, and Boat.

## Histogram analysis

Histogram analysis evaluates the distribution of pixel intensities in encrypted images. For a secure image encryption system, the ciphertext histogram should approximate a uniform distribution, indicating the absence of visually exploitable statistical patterns^38^.

**Fig. 6:**
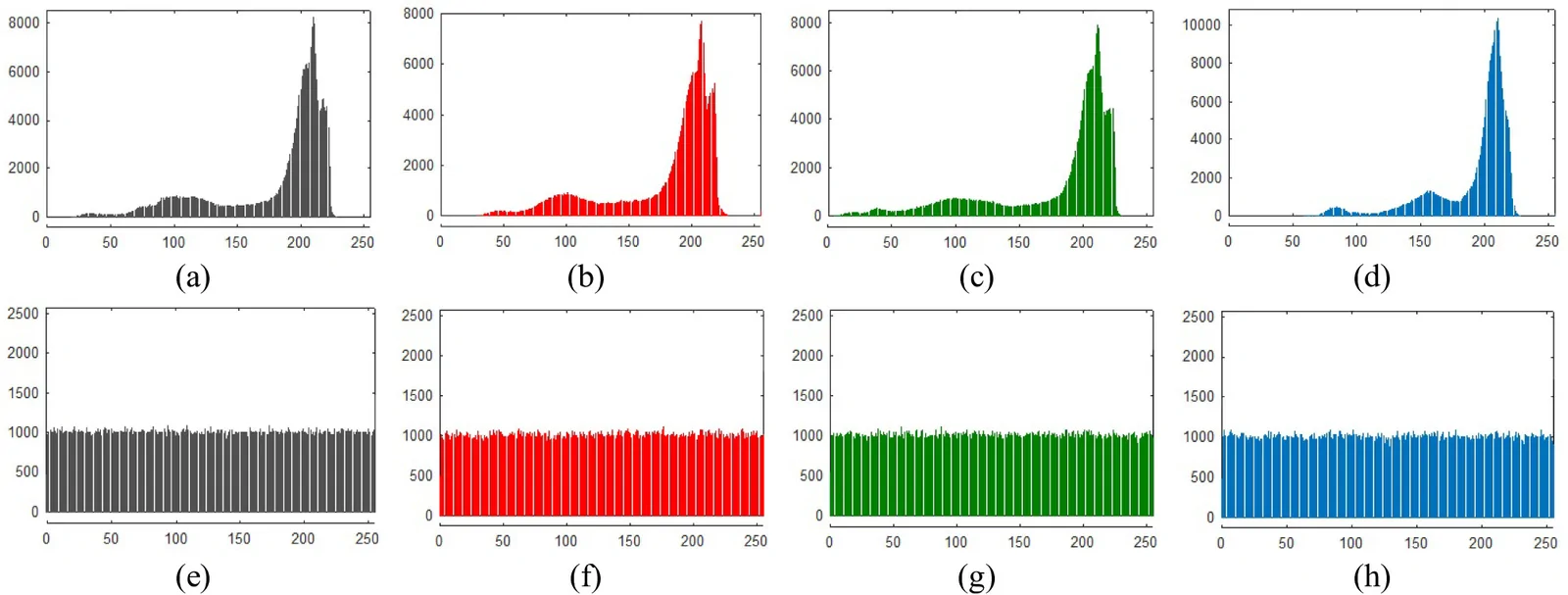
Layer-wise histograms of airplane image. (a) Histogram of source image; (b-d) Histograms of extracted RGB layers; (e) Histogram of Concatenated encrypted RGB layers; (f-h) Histograms of Encrypted RGB layers.

Figure 6 presents the histograms of both source and encrypted images. Unlike the source images, which exhibit structured and content-dependent distributions, the encrypted images display nearly uniform histograms across all intensity levels. This uniformity indicates effective disruption of statistical redundancies present in the plaintext.

It is important to note that histogram uniformity does not constitute formal security proof, rather, it demonstrates that the scrambling and encryption layers remove visually observable intensity patterns that could otherwise facilitate statistical inference.

## Chi-square analysis

To quantify histogram uniformity statistically, beyond the visual assessment, a chi-square goodness-of-fit test was applied to the ciphertext histograms. For an image of MN pixels over 256 intensity levels, the test statistics are:

10 $$\:{\chi\:}^{2}={\sum\:}_{i=0}^{255}\frac{{\left({O}_{i}-{E}_{i}\right)}^{2}}{{E}_{i}},$$

where $\:{O}_{i}$ is the observed frequency of intensity level * i* and $\:{E}_{i}$ = MN/256 is the expected frequency under a uniform distribution. At a significance level $\:\alpha\:=0.05$ with 255 degrees of freedom, the critical value is $\:{{\chi\:}^{2}}_{0.05,\:\:255}\approx\:293.25$; a ciphertext histogram is consistent with uniformity when the computed statistic falls below this threshold. As reported in Table 4, the encrypted images yield chi-square values well below the critical value, confirming that the near-uniform appearance of the histograms in Fig. 6 is statistically significant rather than merely visual.

## Entropy analysis

Entropy quantifies the randomness of pixel intensity distributions. For an 8-bit grayscale image, the ideal entropy value is 8 bits. The entropy of an image is computed as:

11 $$\:H=-\sum\:p\left(x\right){{log}}_{2}p\left(x\right),$$

where $\:p\left(x\right)$ denotes the probability of pixel intensity * x*. Table 5 reports entropy values for the tested images. The encrypted images achieve entropy values close to 8 (e.g., 7.999x), indicating near-maximal uncertainty in pixel distribution. Compared with existing approaches, the proposed method achieves comparable or slightly improved entropy values. These results confirm that the encryption process produces ciphertext distributions consistent with high randomness, reducing susceptibility to entropy-based statistical distinguishers^23,39^.

## Correlation analysis

**Fig. 7:**
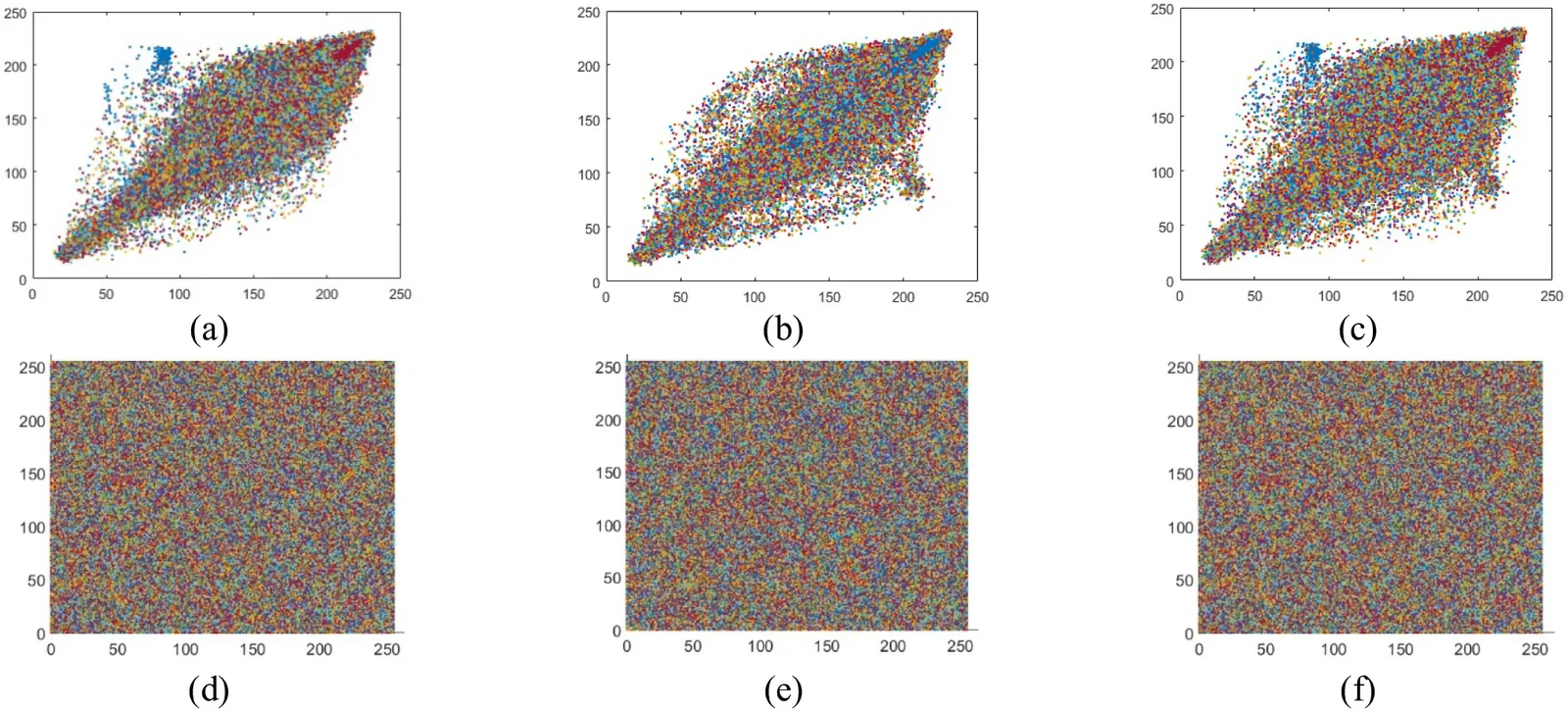
Correlation analyses of adjacent pixels in horizontal, vertical, and diagonal directions. (a-c) correlation analysis of source image; (d-f) correlation analysis of encrypted image.

Natural images exhibit strong correlations between adjacent pixels in horizontal, vertical, and diagonal directions. Effective image encryption should eliminate these correlations^40,41^. The correlation coefficient between two adjacent pixels $\:X\:\mathrm{a}\mathrm{n}\mathrm{d}\:Y$ is computed as:

12 $$\:\rho\:=\frac{Cov\left(X,Y\right)}{{\sigma\:}_{X}{\sigma\:}_{Y}},$$

where * Cov* denotes covariance and $\:\sigma\:$ denotes standard deviation. Table 5 summarizes the correlation coefficients for plaintext and encrypted images. Plain images exhibit high correlations (typically * >0.9*), while encrypted images show correlation values close to zero in all directions. The visual results in Fig. 7 indicate that adjacent-pixel dependencies are effectively destroyed by the scrambling and encryption layers. The near-zero correlation values demonstrate strong spatial diffusion, consistent with expected behavior of secure image encryption systems.

**Table 4:** Chi-square test for ciphertext histogram uniformity (255 degrees of freedom).

Image	$\:{\boldsymbol{\chi\:}}^{2}$ (encrypted)	Critical value $\:\left(\boldsymbol{\alpha\:}=0.05\right)$	Result
Airplane	247.8	293.25	Pass
Baboon	239.1	293.25	Pass
Pepper	251.6	293.25	Pass
House	241.4	293.25	Pass
Boat	243.8	293.25	Pass

**Table 5:** Statistical Evaluation – Entropies and Correlation coefficient analyses.

Entropy analysis					Correlation coefficient analyses				
Image	Plain	Encrypted	Ref. [39]	Ref. [23]	Direction	Plain	Encrypted	Ref. [40]	Ref. [41]
Airplane	6.6879	7.9994	7.9967	7.9989	Horizontal	0.9662	0.0012	−0.0022	0.0009
					Vertical	0.9639	−0.0019	0.0036	−0.0031
					Diagonal	0.9368	−0.0026	−0.0028	0.0040
Baboon	7.7666	7.9997	7.9970	7.9973	Horizontal	0.8534	0.0013	0.0001	0.0072
					Vertical	0.7598	0.0006	−0.0001	−0.0005
					Diagonal	0.7300	−0.0019	0.0045	0.0065
Pepper	7.5835	7.9993	7.9965	7.9977	Horizontal	0.9814	−0.0021	−0.0004	0.0010
					Vertical	0.9833	0.0014	0.0031	−0.0024
					Diagonal	0.9665	0.0009	−0.0031	−0.0067
					Horizontal	0.9664	−0.0012	**–**	−0.0005
House	7.2086	7.9994	**–**	7.9977	Vertical	0.9349	−0.0029	**–**	−0.0051
					Diagonal	0.9122	−0.0008	**–**	−0.0014
					Horizontal	0.9381	−0.0023	0.0005	**–**
Boat	7.1914	7.9994	7.9968	**–**	Vertical	0.9729	0.0008	0.0015	**–**
					Diagonal	0.8944	0.0016	−0.0014	**–**

## Similarity and difference metrics

To further quantify structural dissimilarity between plaintext and ciphertext images, several similarity and difference metrics were evaluated.

- *Structural Similarity Index (SSIM)*: It evaluates the similarity between two images considering luminance, contrast, and structure^42^.

13 $$\:SSIM\left(x,y\right)=\frac{(2{\mu\:}_{x}{\mu\:}_{y}+{C}_{1})(2{\sigma\:}_{xy}+{C}_{2})}{({\mu\:}_{x}^{2}+{\mu\:}_{y}^{2}+{C}_{1})({\sigma\:}_{x}^{2}+{\sigma\:}_{y}^{2}+{C}_{2})}\:,$$

where $\:{C}_{1}$ and $\:{C}_{2}$ are constants for stability, $\:{\mu\:}_{x}$ and $\:{\mu\:}_{y}$ are means of compared images *x* and *y*, $\:{\sigma\:}_{x}$ and $\:{\sigma\:}_{y}$ are standard deviations, and $\:{\sigma\:}_{xy}$ is the covariance of *x* and *y*. Encrypted images exhibit SSIM values close to zero, Table 6, indicating negligible structural similarity with the original images.

- *Normalized Cross-Correlation (NCC)*: It quantifies the similarity between two images by measuring the cross-correlation between their pixel values^7^.

14 $$\:NCC\left(x,y\right)=\frac{\sum\:\left({x}_{i}-{\mu\:}_{x}\right)\left({y}_{i}-{\mu\:}_{y}\right)}{\sqrt{{\sum\:{\left({x}_{i}-{\mu\:}_{x}\right)}^{2}\sum\:\left({y}_{i}-{\mu\:}_{y}\right)}^{2}}}\:,$$

where $\:{x}_{i}$ and $\:{y}_{i}$ are pixel values at corresponding positions, and $\:{\mu\:}_{x}$ and $\:{\mu\:}_{y}$ are means of images *x* and *y*. Low NCC values in Table 6 demonstrate that encrypted images retain no measurable linear relationship with their corresponding plaintexts.

- *Gradient Similarity Index (GSI)*: GSI evaluates similarity of gradient distributions between images.

15 $$\:GSI\left(x,y\right)=\frac{2{\sigma\:}_{x}{\sigma\:}_{x}+{C}_{3}}{{\sigma\:}_{x}^{2}+{\sigma\:}_{y}^{2}+{C}_{3}}\:,$$

where $\:{\sigma\:}_{x}$ and $\:{\sigma\:}_{y}$ are standard deviations of gradients of images *x* and *y*, and $\:{C}_{3}$ is a constant. Values near zero, in Table 6, indicate loss of structural similarity.

- *Pixel Difference Metrics*: Pixels’ difference analysis assesses the disparity between corresponding pixels in two images and evaluates the performance and effectiveness of various image processing techniques, including encryption schemes^43^. We estimate the pixels’ difference among source and encrypted images by evaluating four pixels’ disparity measures, such as Mean Absolute Error (MAE), Mean Squared Error (MSE), Signal-to-Noise Ratio (SNR), and Peak Signal-to-Noise Ratio (PSNR). MAE estimates the average absolute difference between corresponding pixels in two images and measures the average magnitude of the errors. MSE computes the average of the squared differences between corresponding pixels and emphasizes larger discrepancies more than MAE. PSNR measures the ratio between the maximum possible value of the signal and the noise. SNR complements PSNR by relating the power of the original signal to the power of the distortion introduced by encryption.

16 $$\:MAE\left(x,y\right)=\frac{1}{N}{\sum\:}_{i=1}^{N}\left|{x}_{i}-{y}_{i}\right|\:,$$

17 $$\:MSE\left(x,y\right)=\frac{1}{N}{\sum\:}_{i=1}^{N}{\left({x}_{i}-{y}_{i}\right)}^{2},$$

18 $$\:PSNR\left(x,y\right)=10.{{log}}_{10}\left(\frac{MA{X}^{2}}{MSE\left(x,y\right)}\right),\:\mathrm{a}\mathrm{n}\mathrm{d}$$

19 $$\:SNR\left(x,y\right)=10.{{log}}_{10}\left(\frac{{\sum\:}_{i=1}^{N}{x}_{i}^{2}}{{\sum\:}_{i=1}^{N}{\left({x}_{i}-{y}_{i}\right)}^{2}}\right),$$

where * N* is the total number of pixels, $\:{x}_{i}$ and $\:{y}_{i}$ are pixel values at corresponding positions, and *MAX* is the maximum possible pixel value. High MAE and MSE values combined with low SNR and PSNR values indicate that the energy of the plaintext-ciphertext difference is comparable to or exceeds the signal energy, reflecting strong distortion. Table 6 confirms substantial deviation between original and encrypted images across all tested cases. These metrics collectively demonstrate that the encryption process eliminates perceptual and structural resemblance between plaintext and ciphertext images.

**Table 6:** Statistical Evaluation – Pixels’ similarity, difference, and differential attack analyses.

Pixels’ similarity analysis					Pixels’ difference analysis				Differential attack analysis		
Image	Trial	Proposed	Ref.^7^	Ref.^42^	Trial	Proposed	Ref.^43^	Ref.^44^	Trial	Proposed	Ref.^40^
Airplane	SSIM	0.00241	–	0.00154	MAE	86.05	83.03	83.909	NPCR	99.85	99.62
	NCC	0.0115	–	0.0032	MSE	9876.7	10334.5	10962.43	UACI	33.48	33.46
	GSI	0.0016	–	0.0022	PSNR	7.9341	7.99	7.731			
					SNR	1.3	–	–			
Baboon	SSIM	0.00125	0.00161	0.00171	MAE	83.63	76.42	60.693	NPCR	99.79	99.61
	NCC	0.0013	0.0038	0.0029	MSE	9714.74	8631.51	5590.816	UACI	33.45	33.46
	GSI	0.0018	0.0016	0.0031	PSNR	7.8952	8.7833	10.656			
					SNR	0.9	–	–			
Pepper	SSIM	0.00107	0.00113	0.0018	MAE	86.51	81.91	80.159	NPCR	99.81	99.61
	NCC	0.0021	0.0035	0.0025	MSE	9993.06	10036.4	10200.41	UACI	33.52	33.46
	GSI	0.0019	0.0029	0.0038	PSNR	7.9625	8.1666	8.044			
					SNR	1.5	–	–			
House	SSIM	0.00126	0.00124	0.00137	MAE	82.25	–	70.566	NPCR	99.84	–
	NCC	0.0014	0.0018	0.0046	MSE	9342.16	–	7855.912	UACI	33.41	–
	GSI	0.0016	0.0019	0.0026	PSNR	8.1104	–	9.178			
					SNR	1.2	–	–			
Boat	SSIM	0.00117	0.00126	0.00119	MAE	84.34	–	–	NPCR	99.89	99.61
	NCC	0.0027	0.0035	0.0032	MSE	9461.34	–	–	UACI	33.48	33.46
	GSI	0.0018	0.0018	0.0017	PSNR	7.9518	–	–			
					SNR	1.1	–	–			

## Differential attack analysis

Differential analysis evaluates sensitivity of ciphertext to minor changes in plaintext. It involves measuring the impact of altering one or more bits of the input on the resulting ciphertext^40^. We evaluated this analysis on various cipher images under a 1-bit alteration using two common metrics as follows:

- *Number of Pixel Change Rate (NPCR)*: NPCR quantifies the percentage of pixel changes in the ciphertext when a single pixel in the plaintext is altered, indicating the sensitivity of the encryption algorithm to small input changes.

20 $$\:NPCR=\frac{N-M}{N}\times\:100\%\:,$$

where *N* is the total number of pixels in the image and *M* is the number of pixels that remain unchanged in the ciphertext when one pixel in the plaintext is altered.

- *Unified Average Changing Intensity (UACI)*: UACI estimates the average intensity of pixel changes when one bit is altered in the plaintext. It provides a measure of how much the image changes in response to small modifications in the input.

21 $$\:UACI=\frac{1}{N}{\sum\:}_{i=1}^{N}\frac{\left|{C}_{i}-{C}_{i}^{{\prime\:}}\right|}{L}\times\:100\%\:,$$

where $\:{C}_{i}$ and $\:{{C}_{i}}^{{\prime\:}}$ are the intensity values of the *i*^th^ pixels in the original and modified ciphertexts, and *L* is the maximum possible pixel intensity value.

Table 6 reports NPCR values above 99% and UACI values around 33% for all test images. These values are consistent with theoretical expectations for strong diffusion in 8-bit image encryption systems^45^. High NPCR and appropriate UACI values indicate that small perturbations in plaintext result in widespread changes across ciphertext, reflecting strong avalanche behavior. The consistency of the entropy, correlation, similarity, difference, and differential metrics across all five images, spanning low-texture (Airplane), structured (House), and high-texture (Baboon) content, confirms that the scheme generalizes across diverse image types rather than being tuned to a particular class of content.

##  Effect of rounds

The number of scrambling rounds *R* governs the trade-off between diffusion strength and computational cost (Sect. 3.2). To determine an appropriate value, entropy, mean absolute adjacent-pixel correlation, NPCR, UACI, and encryption time were measured as *R* varied on a representative image. As reported in Table 7, the diffusion metrics improve sharply from *R* = 1 to *R* = 2 and saturate at *R* = 4. Entropy stabilizes at 7.999, adjacent-pixel correlation remains near zero, and NPCR and UACI converge to their ideal values, while encryption time grows approximately linearly with *R*. The value *R* = 4 was therefore selected, since additional rounds yield negligible statistical improvement at increasing computational cost.

**Table 7:** Effect of the number of scrambling rounds *R* on diffusion metrics and encryption time (Airplane, 256 × 256).

*R*	Entropy	|Correlation|	NPCR (%)	UACI (%)	Enc. time, 256² (ms)
1	7.9974	0.0123	99.41	31.82	8.4
2	7.9991	0.0041	99.74	33.18	10.3
4	7.9994	0.0015	99.85	33.48	14.2
8	7.9994	0.0014	99.85	33.49	22.0

### Robustness analysis

Robustness analysis evaluates the behavior of the image transformation layer under imperfect transmission conditions, such as noise corruption or partial data loss. It is important to clarify that the proposed framework employs an AEAD scheme, which enforces strict ciphertext integrity verification. Consequently, any modification of the transmitted ciphertext results in authentication failure and decryption terminates. Therefore, the robustness evaluations presented in this subsection are conducted at the scrambling-layer level, prior to AEAD authentication, to assess the intrinsic resilience of the diffusion and scrambling operations rather than the cryptographic security guarantees of the complete system. The practical relevance of this layer-level analysis is twofold. First, it characterizes the information-distribution property of the spin-matrix diffusion by quantifying how localized corruption or loss propagates through the transformed representation. This is the basis on which the broader image-encryption literature reports noise and occlusion behavior, enabling direct comparison with such schemes. Second, it informs deployment variants in which the scrambling layer is applied independently of authenticated encryption, such as perceptual or format-preserving settings where bitstream-level integrity is not imposed. In the complete framework as specified, AEAD integrity ensures that any corrupted ciphertext is rejected prior to decryption. The results below should therefore be interpreted as a characterization of the diffusion transform rather than as end-to-end fault tolerance of the deployed system.

## Noise attack analysis

Noise attacks simulate practical transmission distortions in which encrypted image data is corrupted by random perturbations^46^. To evaluate robustness against such disturbances, Gaussian noise is introduced into the transformed image representation according to:

22 $$\:{I}_{ns}={I}_{org}+G\:,$$

where $\:{I}_{org}$ denotes the original transformed image, $\:{I}_{ns}$ denotes the noisy image, and * G* represents additive Gaussian noise. The objective of this analysis is to examine how the scrambling layer behaves when small perturbations are introduced. Error metrics including MSE and PSNR are computed under varying normalized noise intensities (0.000001, 0.000003, 0.000005, and 0.000007). The corresponding results are summarized in Table 8.

The results indicate only gradual variations in MSE and PSNR as noise intensity increases, suggesting that the scrambling process maintains stable diffusion characteristics under moderate noise conditions. This behavior demonstrates that the transformed image structure does not collapse under small perturbations, which is desirable for practical communication environments where channel noise may be present.

## Occlusion attack analysis

**Fig. 8:**
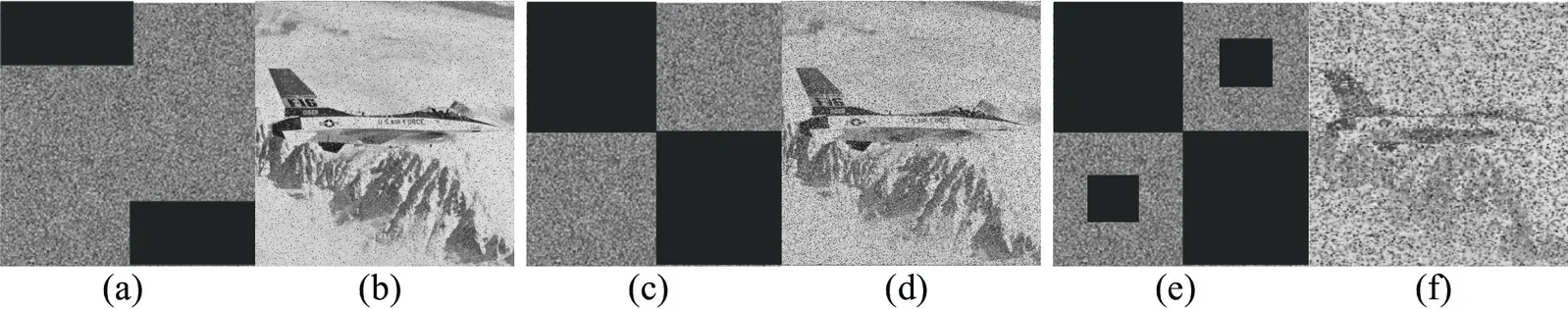
Occlusion analysis for the encrypted Airplane image. (a-b) Occluded by fraction of 1/4 and corresponding recovered image; (c-d) Occluded by fraction of 1/2 and corresponding recovered image; (e-f) Occluded by fraction of 3/5 and corresponding recovered image.

Occlusion attacks model scenarios in which portions of image data are lost or hidden during transmission or storage^46^. The occluded image is modeled as:

23 $$\:{I}_{oc}={I}_{org}\odot\:B\:,$$

where $\:{I}_{oc}$ represents the occluded image and * B* is a binary mask defining the occluded regions. Experiments were conducted by occluding fractions of the transformed image corresponding to 1/4, 1/2, and 3/5 of the total content. Table 8 summarizes the resulting MSE and PSNR values, while representative visual results are illustrated in Fig. 8.

The results show progressive degradation as the occlusion level increases. However, meaningful image reconstruction remains feasible even under significant partial loss (up to approximately 50% occlusion). This indicates that the scrambling process distributes image information across blocks in a manner that mitigates localized data loss.

### Comparative evaluation

To further assess the effectiveness of the proposed framework, comparative analysis was conducted against representative state-of-the-art image encryption schemes reported in the literature. The comparison considers both quantitative performance indicators and qualitative design characteristics, including security assumptions, structural complexity, and practical applicability.

**Table 8:** Error estimation under Gaussian noise and occlusion attack analyses for the encrypted Airplane image.

Image	Trial	Error estimation – Noise intensity				Occluded images		
Airplane		0.000001	0.000003	0.000005	0.000007	1/4	1/2	3/5
	MSE	9811.2	9724.6	9604.3	9502.7	5323.92	3856.75	2108.15
	PSNR	7.9835	7.9927	8.0925	8.2011	11.5201	13.6562	15.0204
Baboon	MSE	9654.8	9521.5	9401.8	9245.2	4809.71	2988.72	2201.01
	PSNR	7.8998	7.9514	8.1263	8.2419	10.9885	12.9899	14.0121
Pepper	MSE	9901.1	9798.7	9612.4	9418.3	5011.07	3121.71	2301.04
	PSNR	7.9818	8.0121	8.2201	8.3611	10.6198	13.0122	14.9811
House	MSE	9244.26	9107.85	9001.22	8892.07	5016.88	3108.74	2094.65
	PSNR	8.1287	8.4587	8.6818	8.8104	11.1416	12.8996	14.9152
Boat	MSE	9397.41	9302.32	9196.74	9056.48	4956.45	3226.41	1994.37
	PSNR	7.9602	7.9698	7.9802	7.9996	10.6553	12.5482	14.3591

## Statistical performance comparison

Tables 4, 5 and 6 present chi-square test for ciphertext histogram uniformity, comparisons of entropy, correlation coefficients, similarity metrics, and differential attack indicators with existing methods. The proposed scheme achieves entropy values close to the ideal value of 8 for 8-bit images, indicating strong randomness characteristics comparable to or slightly exceeding several recent approaches.

Similarly, correlation analysis demonstrates near-zero correlation among adjacent pixels in encrypted images, confirming effective disruption of spatial dependencies. Differential metrics such as NPCR and UACI remain consistent with theoretical expectations for secure image diffusion, showing performance competitive with contemporary schemes.

While numerical differences between methods are often marginal, the proposed framework maintains stable performance across multiple image types, including highly textured and structurally complex content, which indicates robust diffusion behavior.

To position the framework against recent developments, the comparison Table 9 contrasts it with representative image encryption schemes published between 2024 and 2026, spanning chaos-based, quantum-inspired, and post-quantum designs. Beyond the statistical indicators already detailed in Sect. 4.3, the comparison highlights two differentiators. First, the basis of key establishment, whereas schemes described as post-quantum or quantum-inspired predominantly rely on quantum key distribution or chaotic dynamics, the proposed design grounds key establishment in the standardized IND-CCA-secure lattice KEM sntrup761, providing a concrete post-quantum guarantee rather than a heuristic one. Second, encryption efficiency, the measured encryption time for a $\:256\times\:256$ image is competitive with chaos-based schemes that require iterative map evaluations, reflecting the lightweight block-level scrambling and stream-cipher-based authenticated encryption.

## Structural and algorithmic comparison

Beyond statistical indicators, significant differences exist in the underlying design principles among existing approaches. Many reported image encryption schemes rely primarily on chaotic maps, optimization heuristics, or transformation-based operations to achieve randomness^12,19,23^. Although these methods often demonstrate favorable statistical properties, they typically lack formal cryptographic security guarantees and depend heavily on parameter tuning or initial conditions.

In contrast, the proposed framework adopts a layered architecture in which post-quantum key establishment is provided by a standardized lattice-based KEM, authenticated encryption guarantees confidentiality and integrity, and the spin-matrix scrambling layer operates solely as a deterministic diffusion enhancement. This separation between cryptographic hardness and image transformation distinguishes the proposed method from many existing schemes, where scrambling operations implicitly serve as the primary security mechanism.

**Table 9:** Comparison with recent (2024–2026) image encryption schemes.

Scheme	Year	Security basis	PQ key establishment	Entropy	NPCR (%)	Enc. time, 256² (ms)
Proposed	–	Lattice KEM (sntrup761) + AEAD	Standardized lattice	7.9994	99.85	14.2
Alexan et al.^39^	2025	Chaos + S-box	None	7.9967	99.60	160.2
Hwang et al.^42^	2024	Billiard quantum chaos	None (heuristic)	7.999	99.81	–
Yogi et al.^41^	2026	Rule-60 cellular automata + sine map (IoT)	None	7.996	99.62	230
Ding et al.^47^	2025	Multi-wing hyperchaotic + SHA-256	None	7.9993	99.61	345
İnce et al.^48^	2025	Chaotic maps (Tent + Logistic), RSP confusion	None	7.9914	99.61	25

## Post-quantum security perspective

A key advantage of the proposed framework is its explicit grounding in standardized post-quantum cryptographic primitives. Many existing image encryption approaches focus primarily on statistical properties without addressing long-term security against quantum-capable adversaries. By incorporating the IND-CCA-secure sntrup761 KEM together with AEAD-based authenticated encryption, the proposed system ensures that security guarantees originate from well-established cryptographic assumptions rather than solely from image-dependent transformations. Consequently, the framework remains aligned with emerging post-quantum security requirements while maintaining competitive statistical performance. As established in Sect. 4.1, quantum resistance derives solely from the sntrup761 KEM. The spin-matrix scrambling and XOR operations provide no independent quantum hardness, and the symmetric components retain at least 128-bit post-quantum security under Grover’s algorithm. The framework therefore does not claim quantum resistance for the image-transformation layer.

## Practical considerations

From a practical perspective, the proposed method achieves linear computational complexity with respect to image size and introduces only constant communication overhead beyond the encrypted image itself. Compared with approaches requiring iterative chaotic computations, large optimization procedures, or training-based models^7,49^, the proposed framework offers predictable runtime behavior suitable for desktop-scale deployment. Additionally, the deterministic scheduling of spin matrices enables consistent reproducibility without requiring extensive parameter tuning, which improves implementation stability.

## Conclusion

A post-quantum image encryption framework integrating lattice-based key establishment, key derivation, spin-matrix-driven scrambling, and authenticated encryption into a unified architecture was presented. Unlike many existing image encryption schemes that rely primarily on heuristic transformations, the proposed design explicitly separates cryptographic hardness from lightweight image-processing operations. Post-quantum security derives solely from the sntrup761 key encapsulation mechanism, while the spin-matrix scrambling layer provides spatial diffusion rather than cryptographic hardness. Confidentiality and integrity are enforced by AEAD encryption, which resists adaptive attacks under established cryptographic assumptions. Extensive statistical evaluations, including histogram uniformity, entropy, correlation, and differential analyses, demonstrated strong diffusion characteristics and effective disruption of spatial image dependencies. Robustness experiments further showed that the spin-matrix scrambling layer maintains stable transformation behavior under noise and partial occlusion conditions. Comparative analysis with existing methods confirmed that the proposed framework achieves competitive statistical performance while offering clearer security grounding and scalable computational complexity. Overall, the proposed approach establishes a practical bridge between post-quantum cryptography and image encryption, providing a structured foundation for secure visual data transmission. Future work will investigate optimization of transformation parameters, hardware acceleration, and extension toward real-time multimedia encryption scenarios.

## Data Availability

The data generated, used, and analyzed during the current study are available from the corresponding author on reasonable request.
